# Genome-wide associations for multiple pest resistances in a Northwestern United States elite spring wheat panel

**DOI:** 10.1371/journal.pone.0191305

**Published:** 2018-02-07

**Authors:** Kaori Ando, Sheri Rynearson, Kebede T. Muleta, Jhonatan Gedamu, Bedada Girma, Nilsa A. Bosque-Pérez, Ming-Shun Chen, Mike O. Pumphrey

**Affiliations:** 1 Department of Crop and Soil Sciences, Washington State University, Pullman, Washington, United States of America; 2 Ethiopian Institute of Agricultural Research, Holeta Agricultural Research Center, Holeta, Ethiopia; 3 Ethiopian Institute of Agricultural Research, Kulumsa Agricultural Research Center, Assela, Ethiopia; 4 Department of Entomology, Plant Pathology and Nematology, University of Idaho, Moscow, Idaho, United States of America; 5 United States Department of Agriculture–Agricultural Research Service and Department of Entomology, Kansas State University, Manhattan, Kansas, United States of America; Institute of Genetics and Developmental Biology Chinese Academy of Sciences, CHINA

## Abstract

Northern areas of the western United States are one of the most productive wheat growing regions in the United States. Increasing productivity through breeding is hindered by several biotic stresses which slow and constrain targeted yield improvement. In order to understand genetic variation for stripe rust (*Puccinia striiformis* f. sp. *tritici*), *Septoria tritici* blotch (*Mycosphaerella graminicola*), and Hessian fly (*Mayetiola destructor*) in regional germplasm, a panel of 408 elite spring wheat lines was characterized and genotyped with an Illumina 9K wheat single nucleotide polymorphism (SNP) chip to enable genome-wide association study (GWAS) analyses. Significant marker-trait associations were identified for stripe rust (38 loci), *Septoria tritici* blotch (8) and Hessian fly (9) resistance. Many of the QTL corresponded with previously reported gene locations or QTL, but we also discovered new resistance loci for each trait. We validated one of the stripe rust resistance loci detected by GWAS in a bi-parental mapping population, which confirmed the detection of *Yr15* in the panel. This study elucidated well-defined chromosome regions for multiple pest resistances in elite Northwest germplasm. Newly identified resistance loci, along with SNPs more tightly linked to previously reported genes or QTL will help future breeding and marker assisted selection efforts.

## Introduction

Northern regions of the western United States produced approximately 12,000 metric tons of wheat, Triticum aestivum L., in 2015 [1]. Montana, Washington, and Idaho ranked 3^rd^, 4^th^, and 9^th^ in U.S. production respectively, in 2015. Overall, approximately 30% of spring wheat in the US is produced in this region. Several biotic and abiotic stresses create challenges for producing high quality and high yielding wheat in the Northwest states [2–5]. In each state, spring wheat is often planted adjacent to winter wheat as a part of the cropping system. Additionally, annual wheat-based cropping systems, winter-spring cereal rotations, and no-tillage or minimum tillage production are not uncommon. These cropping systems result in a green bridge or residue bridge for several pests and pathogens. Therefore, breeding for resistance to multiple pests and diseases is necessary for sustainable production. Idaho, Oregon, and Washington are known as high-risk epidemiological areas in the US for stripe rust, caused by *Puccinia striiformis* f. sp. *tritici* Westend (PST), due to normally favorable conditions during the growing season [3]. Several stripe rust epidemiological zones are present in these states, as evident from the occurrence and detection of variable PST races across the Northwest each year. In recent years, severe stripe rust outbreaks have led to significant losses throughout northern wheat production areas in the western U.S.; fungicide application costs were estimated at $40 million in Washington alone in 2011 [6].

Hessian fly (HF) (*Mayetiola destructor* [Say]) is a serious insect pest which can cause severe damage on wheat in the Northwest resulting in significant annual yield and quality losses [5]. Although winter wheat may be injured by HF, the timing of spring wheat establishment coincides with the HF’s life cycle in many areas, leading to greater yield losses. Reduced or no-tillage practices and annual cereal cropping increase the chance of survival of HF [7]. Control of HF with insecticidal seed treatments, modifying planting date, altering tillage practices, and/or crop rotations are weakly effective or limit preferred production practices for various reasons. Deploying cultivars with adequate multiple-pest resistances is the most effective and environmentally sound control measure. Wheat varieties resistant to the HF are increasingly being used in the Pacific Northwest (PNW) [8]. However, the potential for emergence of biotypes capable of attacking resistant cultivars is well documented [5,9] and the rate at which HF populations are able to overcome resistance genes is also concerning. There are 35 designated HF resistance genes (*H* genes) to date, which were derived from wheat, wheat relatives and rye (*Secale cereale*). In a recent survey of virulence to *H* genes using populations of HF collected across the southeastern US, only 4 undeployed genes were found to be widely effective [9]. However, diagnostic single nucleotide polymorphism (SNP) markers that would facilitate introgression and selection of *H* genes. In addition, the *H* genes present in many of the Northwestern wheat lines remain inadequately characterized.

*Septoria tritici* blotch (STB), caused by ascomycete fungus *Mycospharella graminicola*, is another potentially severe foliar disease present in most wheat growing regions worldwide. It is especially problematic in areas with cool and wet spring weather [10,11] and regularly causes losses in parts of Montana, Oregon, and Washington [2]. Fungicides have been heavily used to control STB in areas such as Oregon's Willamette Valley, however, emergence of fungicide-resistant populations are of concern [12]. In drier areas with less severe epidemics in the Northwest, fungicide application may not be economical. Twenty designated *Stb* genes on 14 chromosomes are known to date and most have been mapped in wheat [13]. However, many of these genes remain inadequately characterized [14].

The 9K wheat SNP chip-based assay enables robust and affordable genotyping of wheat [15,16]. This chip contains 9K expressed sequence tag (EST) based SNPs, of which most SNPs originated from a single chromosome, and provides adequate genome-wide coverage to conduct genome-wide association studies (GWAS). The 9K SNP chip has been successfully used for GWAS in a variety of traits such as biotic and abiotic stress resistance and grain yield [17–22]. In this study, a panel of 408 elite spring wheat lines which are well adapted to states in the Northwestern US was phenotyped for the pest and diseases which are problematic in the region, including stripe rust, *Septoria tritici* blotch, and Hessian fly resistance. Further, the panel was genotyped with the wheat 9K SNP chip and GWAS analyses were used to discover marker-trait associations.

## Materials and methods

### Plant materials

A panel of 408 elite spring wheat cultivars and breeding lines from various market classes including 153 lines of hard red spring wheat (HRS), 78 lines of hard white spring wheat (HWS), 145 lines soft white spring wheat (SWS), and 32 lines soft white club spring wheat (SWC) were assembled. These lines were developed by private and public programs in California, Idaho, Montana, Oregon, and Washington (S1 File).

### Genotyping

Genomic DNA for each genotype was extracted using the BioSprint Workstation with the BioSprint 96 DNA Plant kit according to the manufacturer's manual (Qiagen, Valencia, CA). DNA was quantified with the Nanodrop (Thermo Fischer Scientific, Wilmington, DE) and diluted with molecular grade water to 50 ng/μL. Genotyping using the Infinium iSelect 9K wheat SNP chip [15] was completed at the USDA-ARS Genotyping Laboratory at Fargo, North Dakota, USA. Allele calling was performed manually for each SNP with Genome Studio v.2011.1 (Illumina Inc., San Diego, CA) and yielded 7,284 scoreable and mapped SNPs.

Simple sequence repeat (SSR) and Kompetitive Allele Specific PCR (KASP) (LGC, Middlesex UK) markers linked to multiple resistance genes were used to genotype the panel. PCR reactions were performed using an iCycler (Bio-Rad Laboratories, Hercules, CA, USA) thermocycler. For SSR markers, a 12 μl reaction mixture containing 100 ng of template DNA, 1.2 μl 10X PCR buffer with 15 mM of MgCl_2_ (Promega, Madiosn, WI, USA), 1.2 μl dNTP (2 mM) (Sigma Chemical Col, St. Louis, MO, USA), 0.48 μl of 25 mM MgCl_2_, 0.3 μl (10 uM) forward and reverse primers, and 0.6 unit of Taq DNA polymerase (New England Biolab, Ipswich, MA, USA). The conditions for PCR were 5 min denaturation at 94°C, 1 min at 94°C, 1 min at 64.2°C, and 1 min at 72°C for 42 cycles, then a final extension step for 10 min at 72°C. SSR markers were analyzed with an ABI3130xl (Applied Biosystems, Grand Island, NY, USA) and alleles scored with GeneMarker V1.91 (Soft Genetics, College Park, PA, USA). For KASP markers, a 10.1 μl reaction mixture contained 150 ng of template DNA, 5 μl of 2X KASP reaction mix, and 0.14 μl of KASP assay. PCR conditions for KASP markers were 15 min of activation at 94°C, 20 sec at 94°C, 1 min at 65–57°C touchdown dropping 0.8°C per cycle for 10 cycles, then 20 sec at 94°C, 1 min at 57°C for 26 cycles. KASP markers were scored with Light-Cycler 480 (Roche, Indianapolis, IN).

### Population structure

Population structure was characterized using a Bayesian phylogenetic method as implemented in STRUCTURE software [23]. STRUCTURE was run in parallel using the ParallelStructure package in R [24]. Parameter K (number of sub-populations) from 1 to 10 was analyzed 5 times with 50,000 burn-in period and 100,000 Markov chain Monte Carlo replications. Subsequently, Structure Harvester [25] was used to determine the critical K value. Once the K value was determined, CLUster Matching and Permutation Program (CLUMPP) [26] was used to obtain the optimal alignment among the replicates. To estimate the probability that two lines, which are not from the same ancestor and share the same copy of an allele, identity by state (IBS) analysis was implemented in JMP Genomics 6.0 (SAS Institute, Cary, NC) software. Principal component analysis (PCA) was conducted using GAPIT [27].

### Linkage disequilibrium

Linkage disequilibrium (LD) *r*^*2*^ for all marker pairs was calculated by TASSEL v3.0 [28]. Linked loci *r*^*2*^ was plotted against map distance (cM) on all chromosomes. A smooth line was fitted for the plot using the loess (local polynomial regression fitting) function in R program (http://www.r-projet.org). Critical value of *r*^*2*^ was calculated as described in [29]. Briefly, the 95th percentile of the distribution was determined after square root transformed *r*^*2*^ between unlinked markers. The map distance where the loess curve and *r*^*2*^ intersect was set as quantitative trait loci confidence intervals (QTL-CI). QTL-CI and *r*^*2*^ was determined for each subset of the population.

### Association mapping analyses

Genotypes with greater than 10% missing genotype data were removed from the analyses, resulting in a total of 395 genotypes for GWAS of stripe rust and HF resistance. For STB, a subset of the panel consisting 198 genotypes was used for analyses (S1 File). Minor allele frequency (MAF) and missing genotype data thresholds were set to 10% and a total of 6,086 SNPs were used for GWAS analyses of stripe rust and HF (S2 File). For STB, 6,084 SNPs were used.

GWAS analyses were conducted using GAPIT [27]. Kinship derived by GAPIT was included to estimate coefficients of relatedness. Mixed linear models with kinship only, kinship with PCA, and kinship with STRUCTURE (Q) were compared by evaluating the results, QQ plots, and Bayesian information criterion (BIC) value obtained from BIC.model.selection option in GAPIT to determine the most suitable model. The BIC analysis indicated that the kinship only was the best model (data not shown), and was thus used in all the GWAS analysis. Criteria for significant marker-trait association was set for *P*-value <0.001. Further, both Bonferroni correction and false discovery rate (FDR) thresholds were set at *P* < 0.1 and the thresholds were applied to examine the significant marker-trait associations. QTL-tagging SNPs were designated as described by Maccaferri et al. [22]. Briefly, when SNPs significantly associated with a trait were co-locating and/or adjacent in the consensus map and within the QTL-CI, the most significant *P-*value SNPs were chosen as the QTL-tagging SNP.

### Stripe rust screening

In order to evaluate stripe rust resistance, the panel was planted in 3 locations in Washington for multiple years: Spillman Agronomy Farm (SP), Pullman, WA (2011, 2012, and 2013), WSU Whitlow Farm (WL), Pullman, WA (2012 and 2013), and WSU Mount Vernon Research and Extension Center (MV), Mount Vernon, WA (2012 and 2013). Resistance was assessed in two locations (SP and WL) in region 1 (R1) [3], which has the greatest number of races and the races with greatest range of virulence, and one location (MV) in region 5 (R5), where mild winters and cool growing seasons result in severe stripe rust epidemics annually. These 7 environments are referred in the text as SP11, SP12, SP13, WL12, WL13, MV12 and MV13, respectively. Five grams of seed for each entry in the panel were planted in non-replicated 1-m single rows with between row spaces 0.25m. Susceptible check Avocet (PI 464644) was planted every twenty plots in WL and MV. In SP, Hank (PI 613585) was planted every 20 rows as a susceptible check. Lemhi (CItr 11415) was planted as a spreader row along the length of the plot every 9 m and surrounding the plots as a border to create adequate disease pressure in SP11-13, MV12, and WL12, and Avocet was used in MV13 and WL13. Seedling infection type (IT) in the field was recorded using a 0–9 scale [30] during tillering stages (Zadoks 20 to 29) due to early uniform epidemic development in SP11, MV12, and MV13. Disease severity (SEV) and IT were measured as described based on the modified Cobb scale [31] up to a maximum of three times at adult stages (Heading to anthesis stages, Zadoks 59 to 70). Data with the greatest distribution of disease scores in each environment were used for GWAS analysis. Disease ratings were collected when the majority of flag leaves of the susceptible checks showed at least 50% of severity (S3 File). Predominant races found in the locations and years tested are described in detail (http://striperust.wsu.edu/races/data/).

### Association mapping validation

An F_5_-derived recombinant inbred line (RIL) population (WA8149/S900001L, n = 273) was used to validate one of the marker-trait-associations (MTAs) detected for stripe rust resistance. Based on pedigree and strong stripe rust resistance phenotype, a SWS breeding line S0900001L (Louise/Zak *Yr5*+*Yr15* SRZ05116//IDO642) was chosen to make a RIL population with a susceptible SWS breeding line WA8149 (Treasure/WA007851//S94148/Wawawai) via single seed descent. The RIL population was planted in SP12 as described above and stripe rust IT was recorded at the adult stage (Zadoks 59 to70). The RIL population was also screened with stripe rust isolates PSTv-14 and PSTv-40 [32] for seedling stage resistance in WSU Wheat Research Facility greenhouse. Eighteen North American differentials were included at each inoculation to confirm the PSTv race [32]. Ten-day-old seedlings were inoculated with PST urediniospores diluted 1:20 with talc powder (Thermo Fischer Scientific). Inoculated seedlings were placed in the dew chamber at 10°C and 100% humidity for 24 hr, then placed in a growth chamber with temperature set for 18°C/day and 6°C/night with 16 h day length. Twenty days post-inoculation, seedlings were scored using IT (0–9 scale) as described above.

Genomic DNA was extracted as described and DNA samples were quantified with Nanodrop and diluted to 50ng/μl. SNP genotyping was performed using Sequenom MassArray System (Sequenom, Inc., San Diego, CA) with 22 markers spanning 22 cM of 1BS based on the consensus map [15]. These SNPs, two *Yr15* KASP markers [33], and one SSR marker (*barc8*) [34] were used to construct a linkage map by JoinMap 4.1 [35]. The Kosambi mapping function and minimum logarithm of the odds (LOD) threshold 3.0 were used. MapChart 2.30 [36] was used to draw the linkage map for QTL.

### Hessian fly (HF) resistance screening

#### Greenhouse experiment with biotype GP

Screening was conducted with HF biotype GP in the greenhouses of the USDA-ARS Hard Winter Wheat Genetic Research Unit greenhouse at Kansas State University, Manhattan, Kansas. Twenty seeds per genotype were sown in flats with 1:1 mixture of soil and vermiculite in greenhouses maintained at 18 ± 3°C with a 14 h light period. Each flat was divided into 22 half-rows. Differential controls which carry known single *H* gene, Ike (*H3*) (PI 574488), 'Caldwell' (*H6*) (Cltr 17897), *H13* and susceptible control, 'Karl92' (PI 564245) were included in each flat. At the one and half leaf stage, seedlings were infested with HF adults under a cheese-cloth cover. Infestation was continued until the egg density reached approximately eight per leaf on average. Three weeks after infestation, phenotypes (susceptible or resistant) were evaluated. Resistant plants grew normally with light green color leaves and dead larvae whereas susceptible plants grew stunted with dark green color leaves with live larvae. Percentage of resistance was calculated as following: number of resistant plants per genotype were divided by the total number of plants examined (S3 File). Genotypes with ≥ 70% resistance were considered resistant.

#### Laboratory experiment with mixed HF biotype

Resistance to a mixed HF biotype population of Northwest origin was evaluated at the University of Idaho, Moscow, Idaho following the method of Schotzko and Bosque-Pérez [37]. Five seeds from each genotype were sown in 10 x 10 cm pots containing a mixture of #1 Sunshine Mix (Sun Gro Horticulture, Agawam, MA), sand, and slow release fertilizer with 5 replications. After germination, plants were thinned to 4 plants per pot. A total of 25 pots were placed randomly in plexiglass cages. In each cage, resistant control with known genes, Ike (*H3*), Caldwell (*H6*), and *H13*, and a susceptible control Karl92, were included. A laboratory colony that has similar frequencies to the biotypes present in fields in northern Idaho and eastern Washington was used for screening. Biotypes GP, E, F and G constitute most of the Hessian fly populations in this region [5]. Ten female and five male HF were released per cage 10 days after planting (2-leaf stage). Twenty-four h after releasing adult HF, presence of eggs on each plant was confirmed. Twenty-one d post infestation, plants were evaluated for presence of larvae and puparia on the primary tiller of each plant. Plants with larvae and puparia were rated as susceptible. Data were converted to percentage of resistance as described above (S3 File).

### *Septoria tritici* blotch screening

A subset of the Northwestern elite spring wheat panel (n = 198, S1 File) was screened for STB in three disease nursery environments in Ethiopia: Holeta in 2013 and 2014 (HT13 and HT14, respectively) and Bekoji in 2014 (BK14). Ethiopia was chosen since it is a hotspot for STB and thus natural inoculum is abundant [11,38]. Each line was planted in 1 m two-row plots with 6 g of seed and a row spacing of 0.2 m. The susceptible spreader 'RB07' (PI 652930) was planted to assess uniformity of disease. The modified Saari-Prescott double digit scoring scale (00–99) was used for evaluating progression of the disease (D_1_) and severity (D_2_) [10,39] (S3 File). D_1_ describes the relative height of the disease progress and D_2_ describes severity measured as disease leaf area. Disease severity percentage was calculated using the following formula [40]:

$$\%\;of\;severity=(D_{1}/9)(D_{2}/9)100$$

Plant height were recorded in HT13 at Zadoks 69 stage.

### Statistical analyses

Analysis of variance (ANOVA) and heritability were analyzed using SAS Proc Mixed procedures (SAS Institute). Pearson correlation-coefficient and Regression analyses were performed using JMP 10.0 (SAS Institute).

## Results and discussion

### Panel population structure and linkage disequilibrium

**Fig 1:**
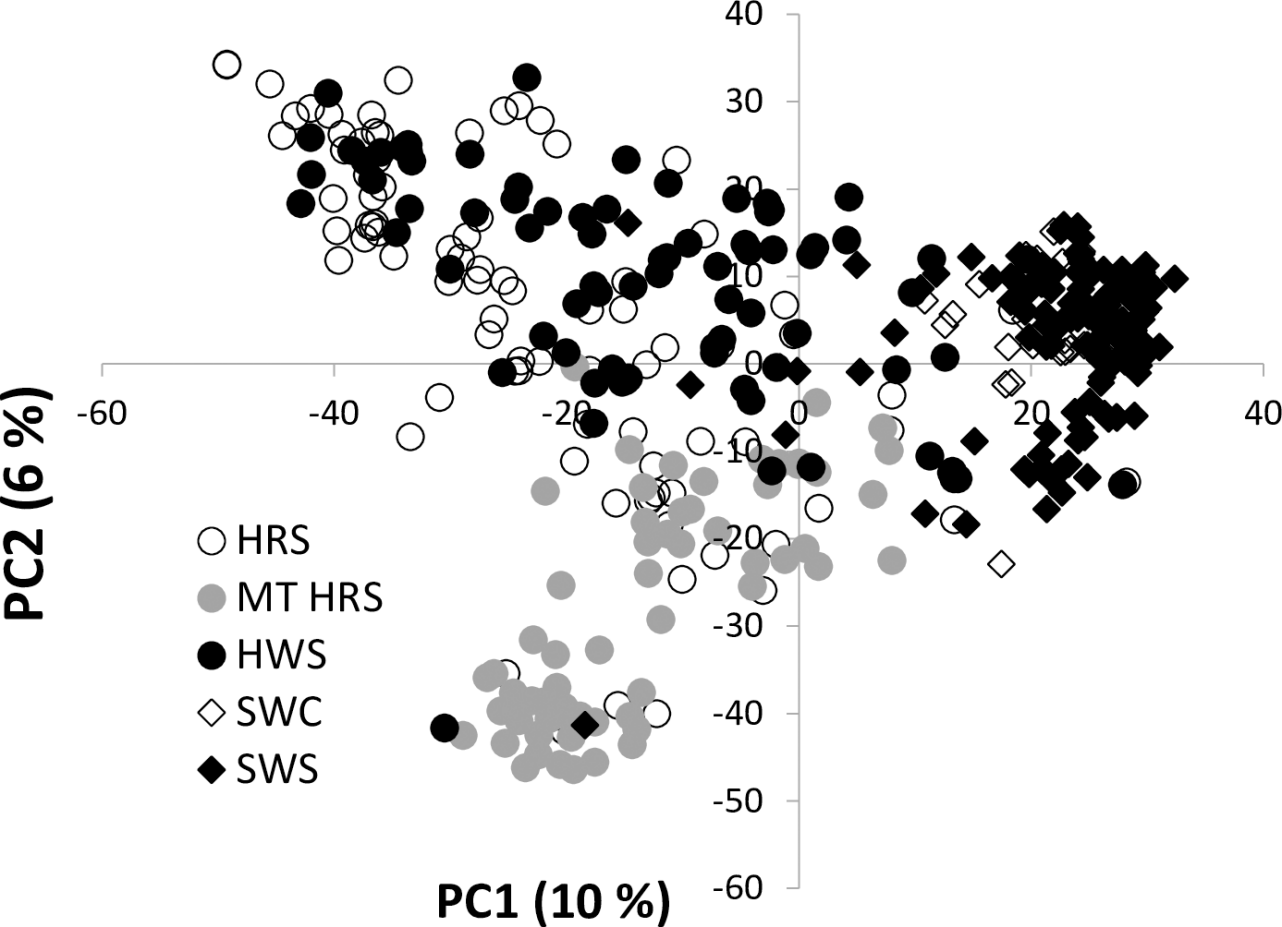
Principal component analysis for the Northwestern elite spring wheat panel.

This panel of elite spring wheat lines from the Northwestern US includes four different market classes of breeding lines and cultivars well adapted to the region (S1 File). Since the presence of population structure can significantly influence GWAS analyses by elevated spurious associations, we examined the population structure with STRUCTURE software [23]. The analysis detected that the likelihood values for K cluster number was 2. The two subgroups obtained by STRUCTURE analysis were divided into primarily soft white spring (51.0%) and hard spring (49.0%) wheat lines (S1 File). Principal component analysis (PCA) indicated there were three groups which are divided into 1) soft white, 2) hard spring wheat from WA, OR, ID, and CA, and 3) hard spring wheat from Montana (Fig 1). Eigenvalues explained PC1 and PC2 at 10% and 6%, respectively. As evident in the population structure analysis, the Northwest elite spring wheat panel population structure was conditioned by two subgroups mainly divided by their major market classes, soft white and hard red wheat. Interestingly however, adding population structure yielded by STRUCTURE or PCA as covariate did not improve the GWAS model. Having the kinship matrix as a random effect in the GWAS model yielded the most powerful analyses for all cases.

**Fig 2:**
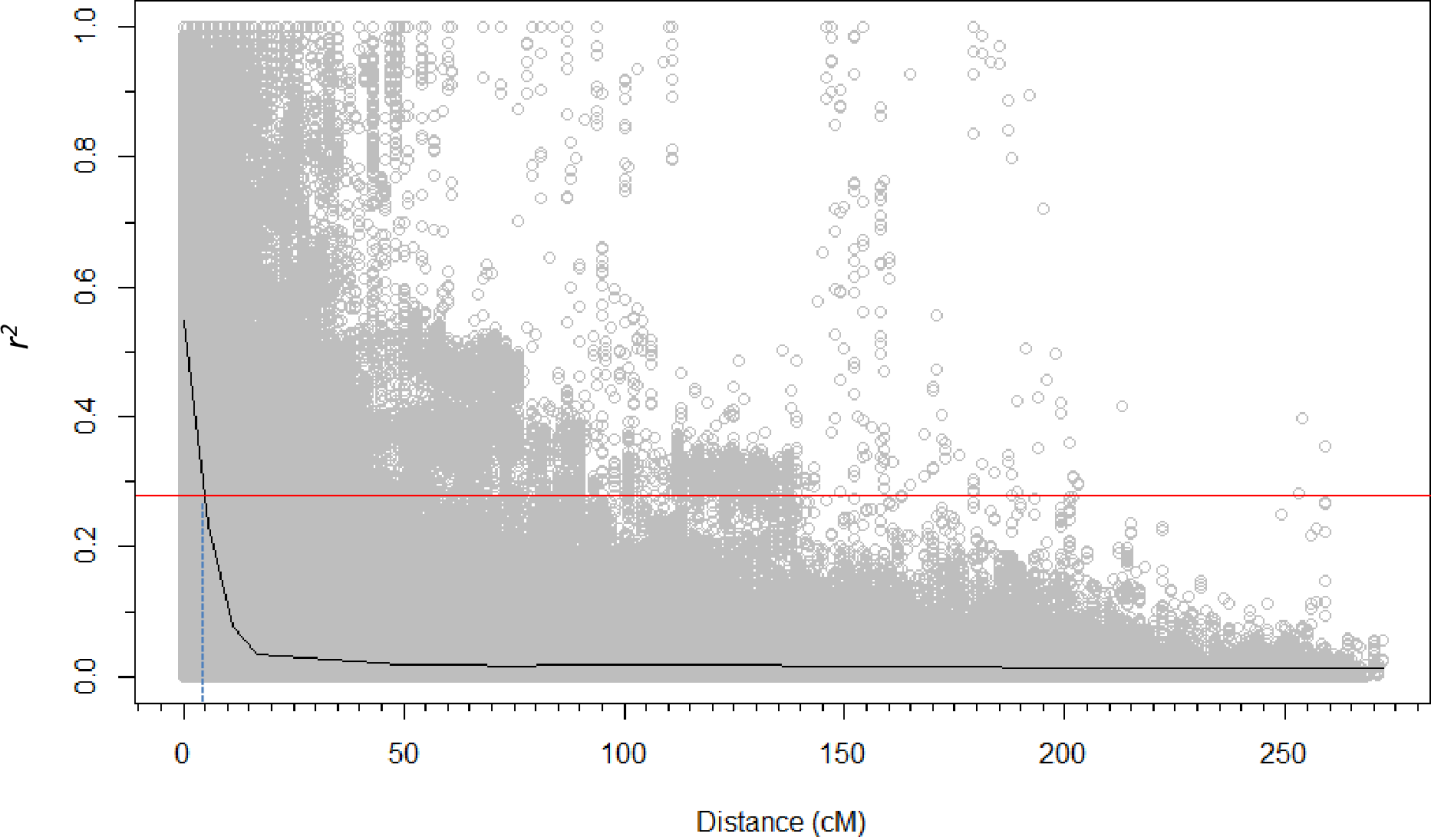
Linkage disequilibrium (LD) decay plot for the Northwest elite spring wheat panel (n = 395). The 95th percentile of *r*^*2*^ (0.28) is shown by the red line. The blue dotted line indicates the intersection between critical *r*^*2*^ and the map distance to determine QTL confidence intervals.

LD was estimated by *r*^*2*^ from all pairs of SNPs on each chromosome for the panel and sub-populations which were used for STB analysis. LD declined to 50% of its initial value at approximately 6 cM for all population sizes. To determine the estimate of the extent of LD to set QTL-CI for each GWAS analysis, the panel and sub-populations were analyzed for LD decay. Critical *r*^*2*^ value ranged 0.28 to 0.30. The intersection with the loess curve and *r*^*2*^ was map distances of 5.1 and 4.5 cM for the panel and subpopulation, respectively (Fig 2).

### Stripe rust resistance

**Fig 3:**
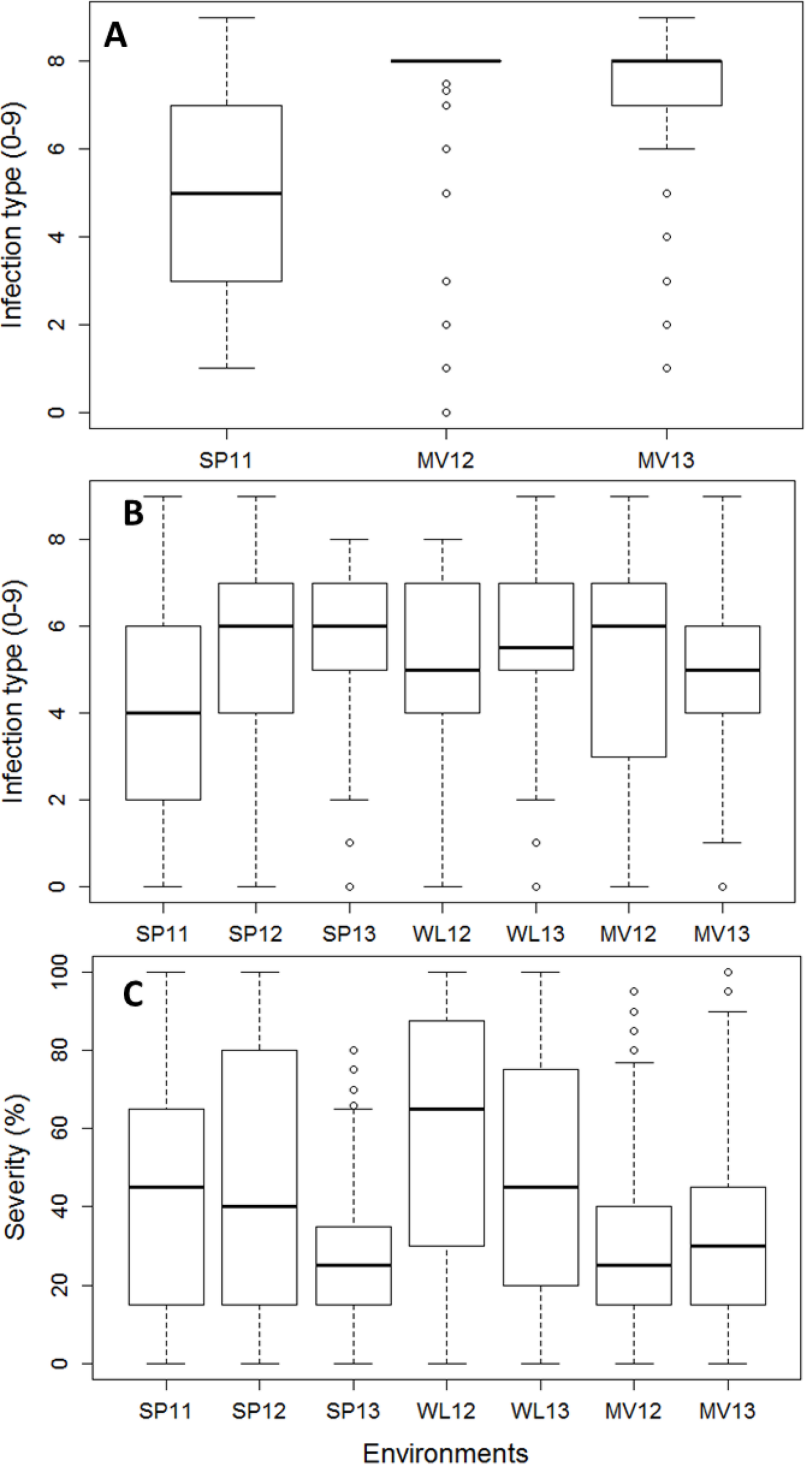
**Boxplot representing the phenotypic distribution for seedling infection type (IT)(A), adult IT (B), and adult disease severity (SEV)(C)**.

Adequate disease pressures were observed in the seven stripe rust screening environments (Fig 3). Predominant stripe rust races were PSTv-4, PSTv-11, and PSTv-14 in R1 and PSTv-11 and PSTv-14 in R5 in 2011 (http://striperust.wsu.edu/races/stripe-rust-race-data.html). In 2012, new races PSTv-37 and PSTv-48 in addition to PSTv-11 were detected in R1 and R5 and it was the same as 2011. In 2013, new races PSTv-52 and PSTv-73 in addition to PSTv-11 and PSTv-37 were detected in R1 location and PSTv-14, PSTv-52, and a new race PSTv-71 were detected in R5. Based on the races in R1 and R5 during 2011 to 2013, there were no races virulent to *Yr5* and *Yr15*.

Pearson-correlation analyses showed that all the traits were significantly correlated in the seven environments (Table 1). The variance components estimates for genotype and genotype by environment interaction were highly significant (*P* < 0.0001) for all locations and across environments (Table 2). The variance components for environment were not significant for each location, however, it was significant (*P ≤* 0.05) across the seven environments. Broad sense heritability estimates (*H*^2^) were calculated with Restricted Maximum Likelihood method [41] which ranged from 0.65 to 0.92. Less environmental variation than genotypic variation yielded high heritability estimates, with non-significant environmental variation estimates and highly significant correlations across the environments.

**Table 1:** Pearson correlation coefficients between stripe rust seedling infection type (IT), adult IT and adult severity in the seven environments.

	Seedling			Adult													
	IT			IT							SEV						
	SP11	MV12	MV13	SP11	MV12	SP12	WL12	MV13	SP13	WL13	SP11	MV12	SP12	WL12	MV13	SP13	WL13
**Seedling IT**																	
**SP11**	1.0																
**MV12**	0.3602***	1.0															
**MV13**	0.4398***	0.5838***	1.0														
**Adult IT**																	
**SP11**	0.4933***	0.3291***	0.3613***	1.0													
**MV12**	0.3848***	0.4003***	0.4807***	0.5903***	1.0												
**SP12**	0.5768***	0.4817***	0.5853***	0.6611***	0.6645***	1.0											
**WL12**	0.5441***	0.4964***	0.5741***	0.6776***	0.6411***	0.7578***	1.0										
**MV13**	0.3554***	0.3784***	0.4808***	0.5775***	0.6358***	0.6535***	0.6557***	1.0									
**SP13**	0.4785***	0.4856***	0.6198***	0.6531***	0.6097***	0.7773***	0.7184***	0.6559***	1.0								
**WL13**	0.4555***	0.4745***	0.6318***	0.6187***	0.6282***	0.7078***	0.6971***	0.6961***	0.7606***	1.0							
**Adult SEV**																	
**SP11**	0.5668***	0.3984***	0.4484***	0.7062***	0.5479***	0.6761***	0.6762***	0.5314***	0.6471***	0.6359***	1.0						
**MV12**	0.2518***	0.2813***	0.3278***	0.4285***	0.5483***	0.5432***	0.4792***	0.6214***	0.4972***	0.5525***	0.4877***	1.0					
**SP12**	0.3079***	0.2415***	0.3240***	0.5287***	0.5403***	0.6487***	0.5431***	0.5553***	0.5399***	0.4856***	0.5085***	0.5910***	1.0				
**WL12**	0.4848***	0.4250***	0.5010***	0.6134***	0.6142***	0.7244***	0.7164***	0.6322***	0.6959***	0.7340***	0.7350***	0.5881***	0.6201***	1.0			
**MV13**	0.3213***	0.3137***	03785***	0.6342***	0.6410***	0.6069***	0.5929***	0.7842***	0.6078***	0.7811***	0.5423***	0.7164***	0.6089***	0.6279***	1.0		
**SP13**	0.4048***	0.3413***	0.4463***	0.5904***	0.5305***	0.6255***	0.6367***	0.6376***	0.6460***	0.6617***	0.5983***	0.6031***	0.6602***	0.6602***	0.6503***	1.0	
**WL13**	0.4402***	0.4007***	0.4744***	0.5828***	0.5646***	0.6366***	0.6071***	0.6568***	0.6467***	0.7811***	0.6433***	0.6105***	0.5046***	0.7709***	0.6805***	0.7068***	1.0

IT–infection type, SEV–severity, MV—Mount Vernon, SP–Spillman, WL–Whitlow, followed by year.

***significant correlation at *P* < 0.001

**Table 2:** Covariance estimates for stripe rust Infection type and disease severity and heritability for 3 locations (MV, SP, and WL) and across environments.

	MV		SP		WL		Across the environments	
	IT	SEV	IT	SEV	IT	SEV	IT	SEV
***σ***^**2**^_***G***_	3.23***	389.84***	3.41***	316.94***	3.10***	722.15***	3.24***	415.94***
***σ***^**2**^_***E***_	0.00^ns^	4.52^ns^	0.68^ns^	136.78^ns^	0.00^ns^	72.4^ns^	0.25*	134.33*
***σ***^**2**^_***GE***_	2.02***	154.47***	1.42***	367.78***	1.13***	229.21***	1.54***	305.04***
***σ***^**2**^_***e***_	0.24**	4.18**	0.30**	5.26**	0.39**	1.00*	0.31***	3.74***
***H***^**2**^	0.74	0.83	0.81	0.65	0.80	0.83	0.92	0.87

MV—Mount Vernon, SP–Spillman, WL–Whitlow, IT—infection type, SEV—severity. *σ*^2^_*G*_ *–*genotype variance estimate, *σ*^2^_*E*_ *–*environment variance estimate, *σ*^2^_*GE*_*−*genotype × genotype variance estimate, *σ*^2^_*e*_ *–*residual variance estimate, *H*^2^ –broad sense heritability

^ns^, *, **, *** nonsignificant, significant at *P* ≤ 0.05, 0.01, or 0.0001, respectively

#### Seedling resistance

To characterize seedling resistance genes present in the panel that are effective against predominant field races of PST, IT data at the seedling stage were recorded in SP11, MV12, and MV13. The panel showed skewed distribution for seedling IT phenotype (Fig 3A). Average seedling IT from three environments showed that 46% of the genotypes had susceptible IT (≥ 7). Less than 10% of the genotypes had resistant IT (≤ 3). Overall mean IT was 6.2, however mean ITs in MV were higher than SP, suggesting adequate disease pressure and virulent races were present from the early season in MV. GWAS analysis for seedling resistance resulted in detection of 44 SNPs on five chromosomes from the three environments (S1 Table). Nine distinct resistance regions were identified based on the QTL-CI of ± 5.1 cM. The most strongly detected resistance loci in SP11 seedling data were on the long arm of chromosome 2B whereas the most significant resistance loci detected in MV12 and MV13 seedling data were on the short arm of chromosome 1B. One SNP, IWA63, which was located on 1BS, was associated with stripe rust resistance in both locations. In fact, IWA63 was detected in all locations and years. More SNPs were significantly associated with seedling resistance in MV (23 SNP) than SP (10 SNP), which is perhaps not surprising since more virulent and diverse PST races are normally detected in eastern WA compared to western WA each year, including the environments in the present study.

#### Resistance in adult stages

The panel exhibited near-normal distributions for the measured traits (Fig 3B and 3C). The mean adult plant IT rating from the seven environments was 4.9. Twenty-four percent of the genotypes had resistant ITs (≤ 3) while 14% of genotypes had susceptible ITs (≥ 7). SP11 had lower mean IT (3.8) than the other environments ranging from 4.8 to 5.3. Mean adult plant SEV from the seven environments was 32.5. Most of the genotypes (91%) had less than 50% SEV phenotype.

GWAS for adult IT reactions identified a total of 114 significant SNPs found on 11 chromosomes from all environments (S2 Table). These SNPs were assigned into 22 distinct regions based on the 5.1 cM QTL-CI. The most significant locus was detected on 1BS. Two loci were commonly found in three locations, SP, MV, and WL, on 1BS. Two loci were found commonly between WL and SP on 1B and 5B and three loci were common in WL and MV on 1B and 2B. By contrast, one common locus on 5A was found between MV and SP.

GWAS for adult SEV phenotypes resulted in the identification of 91 significant SNPs on 11 chromosomes (S3 Table). There were two resistance loci on 1BS which were commonly detected in all three locations. These loci were also commonly identified in the three locations for adult IT phenotype. Two loci on 7A and 7D were common between SP and MV and one locus on 1B was common between WL and MV. However, three loci on 1BS and 2BL were common between SP and WL.

All loci detected in seedling IT, adult IT and adult SEV GWAS were analyzed to evaluate which regions are associated with all-stage resistance or adult plant resistance (S4 Table). Four loci were significantly and commonly associated with stripe rust resistance observed in seedling IT, adult IT, and adult SEV (Table 3). These loci are most likely related to all-stage resistance which manifests effectiveness against stripe rust throughout the growing season. The locations of the QTL were predominantly on 1BS with 3 QTLs and one QTL on 2BL. QTL-tagging SNPs on 1BS, IWA2583 and IWA2561 had *P* values significant at Bonferroni threshold (*P* < 0.1) in at least one environment.

**Table 3:** Chromosome location, previously reported *Yr* genes and QTL, and *R*^*2*^ values of significantly and commonly associated QTL with stripe rust seedling infection type (IT), adult IT and adult severity phenotype.

QTL	*Qyr*.*pnw*.*R-1BS*.*1*	*Qyr*.*pnw*.*R-1BS*.*2*	*Qyr*.*pnw*.*R-1BS*.*3*	*Qyr*.*pnw*.*R-2BL*.*1*
SNP	IWA2583^c^	IWA2561	IWA6450	IWA2702
**Chromosome**^a^ [15]	1B	1B	1B	2BL
**Position (cM)**^a^ [15]	18.1–18.4	23.7–30.5	40.4	195.8
***Yr* genes**^b^ [22,42]	*YrAlp*	*YrAlp*, *Yr15*, *YrH52*, *Yr64*	*Yr24/Yr26*	*Yr53*
**QTL**^b^	*Qyr*.*cau-1BS_AQ24788-53*, *QYr*.*wpg-1B*.*2 (IWA63); QYr*.*wsu-1B*.*1(IWA5963)*	*Qyr*.*caas1BL*.*1RS_SHA3/CBRD;* *QYrco*.*wpg-1B*.*2_Coda;* *QYrwpg-1B*.*3(IWA7578);* *IWA6891_Seedling*		*Qyraq*.*cau-2BL_Aquileja*
**Seedling IT**	Phenotypic variation explained by SNP in percent (*R*^*2*^)			
**SP11**	2.0	0.8	1.0	3.8
**MV12**	3.3	4.9	1.3	0.3
**MV13**	8.5	8.1	2.7	0.9
**Adult IT**				
**SP11**	0.2	0.5	0.4	2.0
**SP12**	3.1	3.3	1.8	1.9
**SP13**	5.6	5.4	1.7	1.5
**MV12**	5.2	3.9	2.5	1
**MV13**	5.2	4.2	1.1	0.3
**WL12**	4.7	2.3	2.3	0.8
**WL13**	6.1	4.6	2.3	1.2
**Adult SEV**				
**SP11**	3.2	1.4	1.1	3.7
**SP12**	2.7	2.5	1.4	1.9
**SP13**	3.6	2.4	0.6	1.0
**MV12**	5.1	2.8	1.5	1.5
**MV13**	2.9	1.5	1.6	1.2
**WL12**	5.4	4.6	1.9	2.3
**WL13**	3.9	2.5	2.3	1.8

IT–Infection type, SEV—severity

^a^Chromosome location

^b^Known *Yr* genes and QTL location

^c^SNPs with bold font were identified significant at Bonferroni *P* ≤ 0.1 in seedling IT, adult IT and adult SEV.

*Qyr*.*pnw*.*R-1BS*.*1* (IWA2583) consists of two SNPs spanning an 18 cM region on the distal end of 1BS. Phenotypic variation explained by IWA2583 was up to 8.5%. Comparison with the consensus map created by Maccaferri et al. [22] revealed that this QTL corresponds with the locations of *YrAlp*, and three QTL: *Qyr*.*cau-1BS_AQ24788-53*, *QYr*.*wpg-1B*.*2*, and *QYr*.*wsu*.*1B-1*. *YrAlp*, an all-stage resistance gene, were isolated from a Washington cultivar Alpowa and is ineffective against the predominant races currently in Washington [43]. Alpowa was included in the panel and average seedling IT score for Alpowa across the environments was 6.7. Therefore, it is unlikely that *Qyr*.*pnw*.*R-1BS*.*1* is detecting *YrAlp*. Interestingly, *QYr*.*wpg-1B*.*2* had both SNPs from *Qyr*.*pnw*.*R-1BS*.*1* in common. However, *QYr*.*wpg-1B*.*2* was detected for seedling stage resistance in a Pacific Northwest winter wheat panel but not in adult stage resistance screening [44]. By contrast, *Qyr*.*pnw*.*R-1BS*.*1* was significantly related to seedling resistance as well as adult plant resistance in this study.

*Qyr*.*pnw*.*R-1BS*.*2* (IWA2561) overlaps with the confidence intervals of *YrAlp*, *Yr15*, *YrH52*, *Yr64*, and four QTL: *Qyr*.*caas1BL*.*1RS_SHA3/CBRD*, *QYrco*.*wpg-1B*.*2_Coda*, *QYrwpg-1B*.*3*, and *IWA6891_Seedling*. *YrH52* is originally derived from H52, a *T*. *dicoccoides* accession [45] and has not been widely used in breeding. Thus, it is unlikely the *Qyr*.*pnw*.*R-1BS*.*2* is *YrH52*. Also, *Yr64* was found in durum wheat, PI 331260, from Shewa, Ethiopia [46]. Introgressing *Yr64* to common wheat for breeding is a recent effort [47], therefore, *Yr64* is not expected in the panel. By contrast, based on the pedigree analysis there are 58 lines in this panel that potentially carry *Yr15*. It is possible that *Qyr*.*pnw*.*R-1BS*.*2* is detecting *Yr15* since none of the PST races detected in the tested environments are virulent to *Yr15*. Thirty-three lines had the resistance-associated haplotype at *IWA2561*, which represents 8.4% of the panel. Markers for *Yr15*, *barc8* [34], KASP-R5 and KASP-R8 [33] were used to screen the panel. Thirty lines were identified by *barc8* as positive for the *Yr15* allele. Twenty-six lines were identified by the flanking KASP-R5/R8 markers. Of the 26 lines, 14 were potentially expected to carry *Yr15* based on pedigree. This marker data was not used for complete GWAS analyses since the MAF cutoff was set at >10%. When using a less stringent MAF >5% threshold, the *Yr15* markers were generally the most significant markers associated with rust resistance (data not shown). Therefore, *Qyr*.*pnw*.*R-1BS*.*2* may be conditioned by *Yr15* and/or additional uncharacterized genes.

*Qyr*.*pnw*.*R-1BS*.*3* (tagged by IWA6450) corresponds with the confidence intervals of *Yr24/26* [46,48]. *Yr24/26* is widely deployed in China, but not in the US. In addition, though the frequency is low, the PST races virulent to *Yr24/26* were detected in R1 region during 2011–2013 and R5 in 2013 (PSTv-40 and PSTv-41). Further characterization of this resistance locus is warranted.

*Qyr*.*pnw*.*R-2BL* (tagged by IWA2702) was the only resistance-associated locus on 2BL located at 195.8 cM. IWA2702 explained 0.3 to 3.8% of the variation. This region corresponds to *Yr53* and resistance locus *Qyraq*.*cau-2BL_Aquileja* [49,50]. *Yr53* was found in durum wheat accession PI 480148, which was recently characterized and has not been used as a parent for any of the lines in the elite Northwest panel. Another *Yr* gene on 2BL, *Yr5*, does not overlap with the confidence interval of *Qyr*.*pnw*.*R-2BL* based on the consensus map. However, based on recently developed markers flanking *Yr5* [44], it was located between 199.3 to 206.2 cM. Given the possibility for variable or longer-range LD in certain germplasm or regions of a chromosome [51], it is not clear whether *Qyr*.*pnw*.*R-2BL*, *Yr53* and *Qyraq*.*cau-2BL_Aquileja*, or *Yr5* represent distinct loci.

Four loci were only associated with seedling IT phenotypes and were not detected in adult plant field resistance screening (S4 Table). These loci were located on 1BS, 2BL, 5AL, and 5BL. Loci on 1BS were located within the QTL-CI either *Qyr*.*pnw*.*R-1BS*.*1* or *Qyr*.*pnw*.*R-1BS*.*3*. Locations for two resistance loci were on 2BL, tagged by IWA8599 and IWA2873, and correspond with the locations of *Yr41* and *Yr53*, respectively. *Yr41* is an all-stage *Yr* resistance gene derived from CN19 [52]. However, *Yr41* is not widely used in the US (X. Chen, unpublished data). Loci on 5AL (IWA7009) and 5BL (IWA6902) correspond with the previously reported QTL, *QYr-5A_Opata85* and *QYr*.*cim-5AL_Pastor* for 5AL QTL; *QYr*.*sun-5B_Wollaroi* and *QYr*.*ui-5B_IDO444* for 5BL [53–56]. However, the previously reported QTL were identified for APR not for seedling resistance. It is possible these loci were not detected in adult stages due to PST races virulent on these genes increasing or arriving later in the field environments. Also, since many lines have APR resistance QTL, they may be masked by other loci with stronger effects.

Twenty-four loci were detected only in adult IT or SEV phenotype analyses based on the QTL-CI (S4 Table). These loci map to 20 chromosome arms: 1BS, 2AS, 2AL, 2BL, 3BS, 3BL, 4AS, 4AL, 4BL, 5AS, 5AL, 5BL, 5DS, 6BS, 6BL, 7AS, 7AL, 7BS, 7BL, and 7DL. In addition, four loci were commonly detected between adult IT and SEV on chromosomes 2BL, 3BS, and 5BL. These resistance-associated loci could be unique adult plant resistance loci. Many of the resistance loci detected correspond with the positions of designated *Yr* genes and previously reported QTL locations. QTL on 1BS (tagged by IWA7578) and 2BL (tagged by IWA2179) were detected at the same positions as *Qyr*.*pnw*.*R-1BL*.*1* and *Qyr*.*pnw*.*R-2BL* [44], respectively. A QTL on 3BS (tagged by IWA5203) corresponded to the positions of *Yr30*, *Yr57*, and many QTL. One of the previously reported QTL, *QYr*.*ucw*.*3B*.*2*, was 0.2 cM distal from IWA5203 [22]. *Yr57* is all-stage resistance gene derived from Australian wheat landrace, AUS27858 [57]. The pleiotropic APR locus *Yr30* is either tightly linked or pleiotropic to stem rust gene *Sr2* and pseudo-black chaff (*Pbc*) [56,58]. One of the *Yr30*-linked markers, *gwm533*, is located 3.9 cM proximal to IWA5203. In addition, *Yr30* is known to be present in some lines in this panel, therefore this locus is likely detecting *Yr30*. A resistance locus on 7BL QTL tagged by IWA4857 was mapped to the same region as *Yr39*. *Yr39* is characterized as a high-temperature adult-plant (HTAP) gene for resistance to stripe rust and derived from Alpowa [43]. This gene is more effective when temperature gradually increases at adult plant stages. Overall average adult IT for Alpowa was 4.1 and it had a resistant IT (< 3) at SP11, MV12, and MV13. As was in the case for *Yr30*, this association is likely detecting *Yr39* since many of the Washington lines in the panel carry *Yr39* and it was effective at the tested environments. A resistance locus on 6BS tagged by IWA1493 corresponded with the *Yr35* region, which is derived from *T*. *dicoccoides* and confers seedling resistance [59,60]. However the closest flanking marker (*cfd1*) was 29 cM away from the QTL based on the consensus map. Resistance loci on 2AL, 5AL, 5BL, 5DS, 6BL, 7AS, and 7BS overlapped with the chromosome locations of previously reported QTL and it is likely these loci are corresponding with the alleles from previously reported QTL. QTL on 4BL (tagged by IWA1798), 6BS (tagged by IWA1493), and the distal end of 5AS QTL (tagged by IWA2145) do not correspond with designated *Yr* genes or previously reported QTL, and could be novel loci for stripe rust APR.

### AM validation

Resistant parent S0900001L had low overall average phenotypes, 1.3, 0.8, and 5.6 in the field observation of seedling IT, adult IT, and adult SEV across the seven environments, respectively. Initially, the RIL population was examined for adult plant resistance in the field. It was segregating 1:1 for susceptible and resistant with a chi-square (χ^2^) value of 4.5 and associated *P*-value of 0.10. The RIL population was also screened with single PST races, PSTv-14 and PSTv-40, for seedling resistance. These PST races contained equivalent virulence to the genes on 1BS and 2BL with the predominant PST races when the data for GWAS were collected. Seedling phenotypes were also segregating 1:1 for resistant and susceptible with *P*-values of 0.16 (PSTv-14) and 0.25 (PSTv-40).

The RIL population was genotyped with 22 SNP markers which were located between 13.2 to 35.3 cM on the 1BS region based on the consensus map developed by Cavanagh et al. [15]. These SNPs spanned the QTL regions for *Qyr*.*pnw*.*R-1BS*.*1* and *Qyr*.*pnw*.*R-1BS*.2 that were identified by the GWAS result. Thirteen SNPs were polymorphic between the parents and grouped in one linkage group. KASP-R5, KASP-R8 and *barc8* markers for *Yr15* were also included to genotype the RIL population to further verify the relationship between the QTL and *Yr15* since S0900001L has *Yr15* in its pedigree.

**Fig 4:**
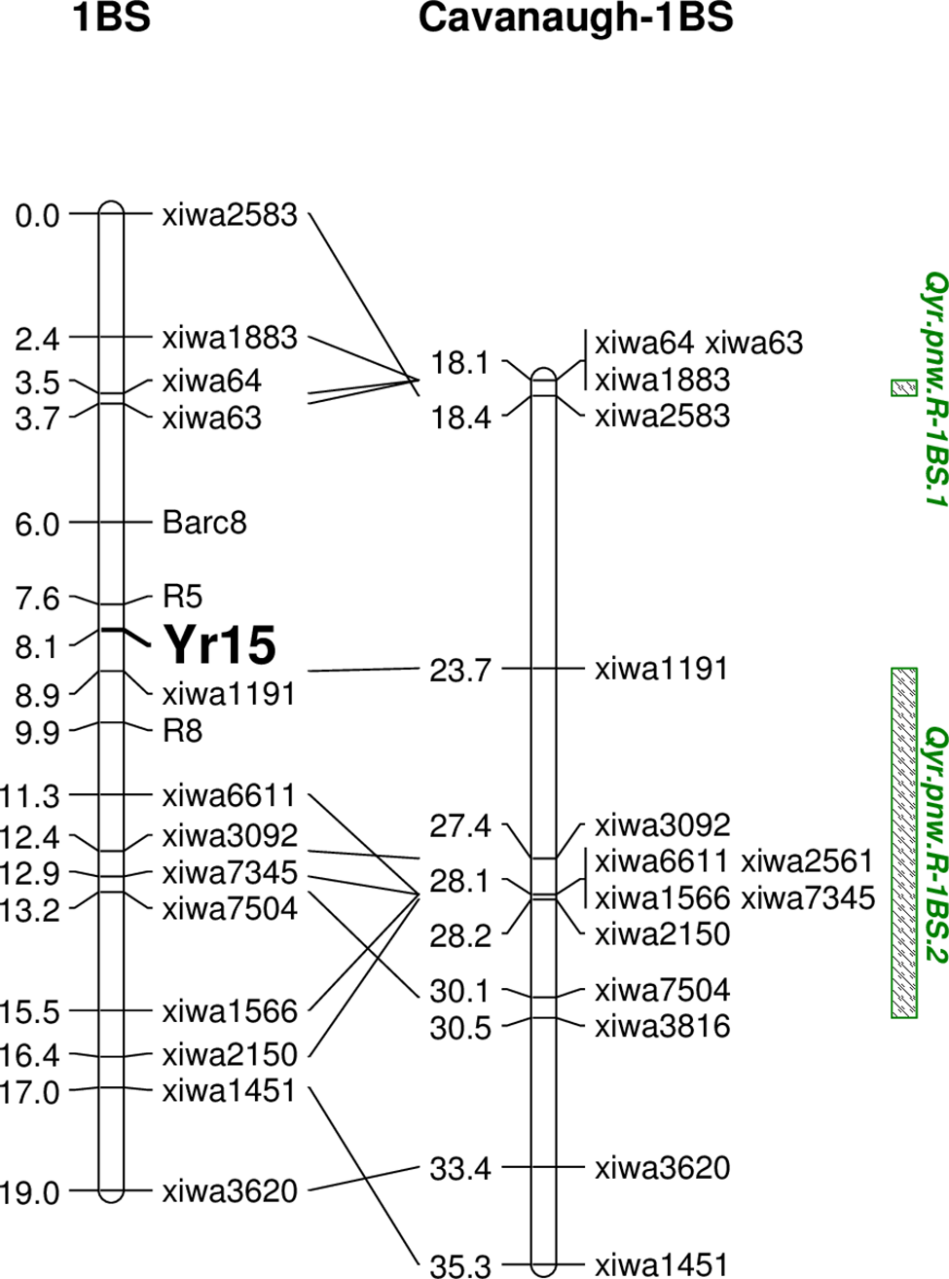
Partial genetic linkage map of chromosome 1BS from WA8149/S0900001L recombinant inbred line population showing *Yr15* location and comparisons with the consensus map of Cavanagh et al. (2013). The lines connect markers in common between the two maps. Green-shaded boxes denote two stripe rust resistance QTL identified from the panel in this study.

The RIL population linkage map order mostly agrees with the 9K consensus map [15], though there were some micro chromosome order differences (Fig 4). *Yr15* was located between flanking KASP markers and IWA1191. Thus, *Qyr*.*pnw*.*R-1BS*.*2* is most likely conditioned by *Yr15*. SNP IWA1191 was located between flanking KASP markers. This RIL mapping further validates the KASP-R5/R8 flanking markers for *Yr15* and detection in this panel.

### Hessian fly resistance screening

**Fig 5:**
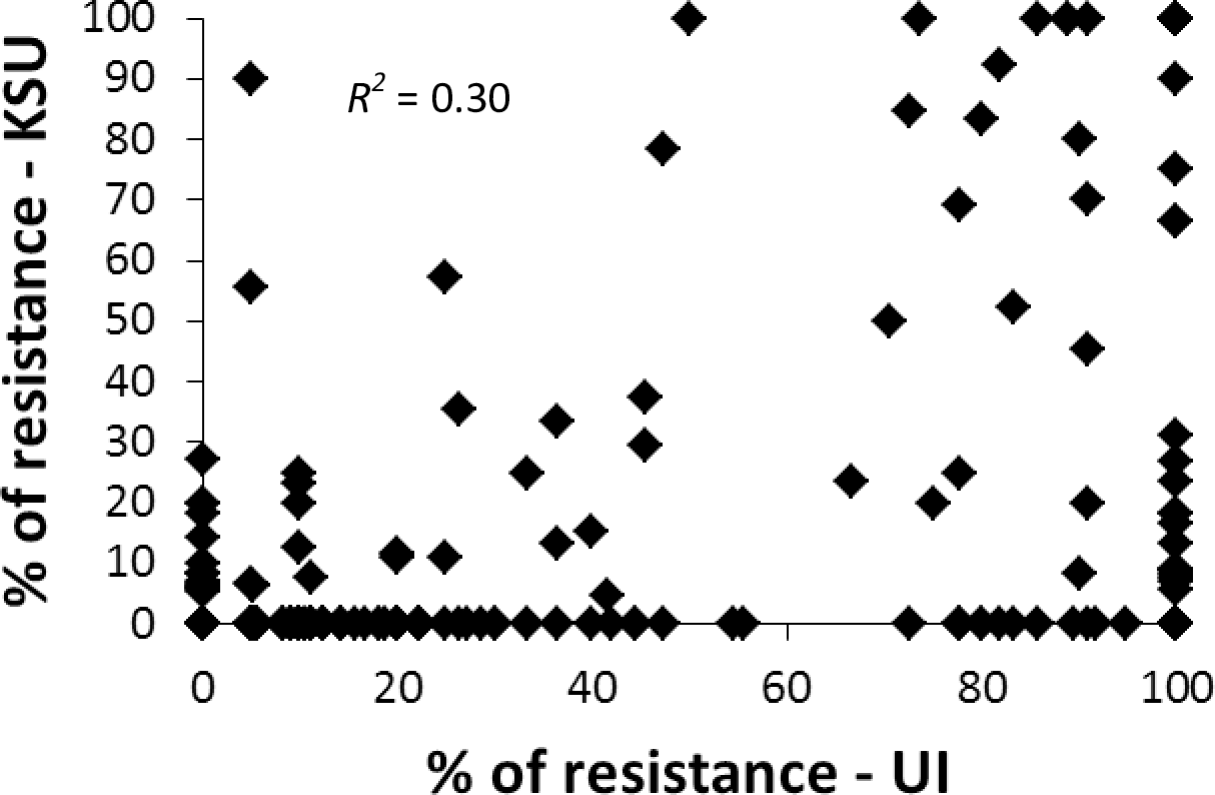
Scatter plot showing percentage of Northwestern elite spring wheat panel lines resistant to infection by two Hessian fly populations. *R*^*2*^ for linear regression.

Screening with a mixed biotype population of HF from Washington and Idaho revealed that 130 (33%) of the 395 lines were resistant. Distribution of the resistant phenotypes was bimodal and skewed towards susceptibility (Fig 5). The majority of the resistant lines were from Idaho, Oregon, and Washington breeding lines, which is consistent with the areas that most commonly experience HF losses. On the other hand, the vast majority of the panel was highly susceptible to the GP biotype screened in Kansas, as was seen in the highly skewed phenotype distribution. Twenty-one genotypes (5%) were identified as resistant to the GP biotype population.

The Pearson correlation between the resistance rating data for both HF populations was 0.30 (*P* < 0.0001) (Fig 5). Eighteen genotypes were resistant to both KSU and UI HF populations (S1 File). Ten lines have the cultivar 'Louise', or one of the parents of Louise, 'Wawawai', in their pedigree. Louise was thought to carry *H3* and *H5* genes based on the pedigree [61]. However, mapping HF resistance from Louise showed that a 1AS resistance locus was not allelic to either *H3* or *H5* [61]. In addition, five lines, IDO584, IDO585, IDO858, IDO868, and UI Cataldo, developed by the University of Idaho share the same *H25* donor parent, KS92WGRC20 [62,63]. Three resistant genotypes developed by WSU, WA008076, WA008101 and Otis had neither Louise nor KS92WGRC20 in their pedigree. Interestingly, WA008101 has Otis in its pedigree, therefore the sources of HF resistance for WA008101 and Otis could be the same. However, their source of HF resistance remains unknown. Many genotypes identified as resistant to the UI HF population were susceptible to the KSU HF population. In addition, a majority of the genotypes resistant to the KSU biotype were also resistant to UI HF population except UC1599. It is unknown which HF resistance gene is present based on the UC1599 pedigree of Summit/3/HAHN/TURACO/2/TURACO.

GWAS analysis of infestation data using only the UI HF population was conducted since the resistance frequency to the KSU HF population was too low for a reliable analysis. Nine QTL on four chromosomes were identified (Table 4). The locus tagged by IWA6803 on chromosomes 6A and 6B was the only significant locus when using the stringent Bonferroni correction threshold (*P* < 0.1). The most consistent resistance-associated loci were detected on chromosomes 6A and 6B.

**Table 4:** Quantitative trait loci (QTL), QTL tagging SNP, chromosome location, *P* and *R*^*2*^ values for UI Hessian fly population.

QTL	SNP	Chr.^a^	Position^a^	*P* value	*R*^*2*^^b^
*QHf*.*pnw*.*2B*	**IWA3982**^**C**^	2B	259.5	1.4E-06	4.2
*QHf*.*pnw*.*4B*	**IWA7854**	4B	67.2	7.4E-04	2.0
*QHf*.*pnw*.*6A*.*1*	**IWA3296**^**C**^	6A	0.9	7.1E-06	3.7
	IWA969^**C**^		0.9	2.2E-04	2.5
*QHf*.*pnw*.*6A*.*2*	**IWA6803**^**C**^		6.2	6.2	14.3
	IWA1254^**C**^		11.1	11.1	2.3
*QHf*.*pnw*.*6A*.*3*	**IWA7457**		25.5	3.1E-04	2.3
*QHf*.*pnw*.*6A*.*4*	**IWA1522**		45.7	8.0E-04	2.0
*QHf*.*pnw*.*6B*.*1 (H34)*	IWA860	6B	0.3	3.1E-04	2.3
	IWA3296^**C**^		0.9	7.1E-06	3.7
	IWA745		0.6	2.8E-05	3.2
	**IWA3982**^**C**^		0.6	1.4E-06	4.2
	IWA969^**C**^		0.9	2.2E-04	2.5
*QHf*.*pnw*.*6B*.*2*	IWA666		2.6	7.9E-07	4.4
	IWA8314		4.2	5.3E-06	3.8
	IWA921		4.8	2.5E-05	3.2
	**IWA6803**^**C**^		6.2	8.6E-18	14.3
	IWA1254		11.1	3.1E-04	2.3
*QHf*.*pnw*.*6B*.*3*	**IWA7841**		15.7	3.4E-07	4.7

UI–University of Idaho, SNPs in bold are QTL-tagged SNP for the loci

^a^SNP location on chromosome and position based on the consensus map by [15]

^b^Phenotypic variation explained by the SNP in percent

^c^SNP has more than two loci

One significant association was identified on the long arm of chromosome 2B, *QHf*.*pnw*.*2B*. There are two *H* genes reported on 2B, *H20* and *H21* [64,65]. *H20* was transferred to wheat from *T*. *durum* cultivar ‘Jori’. There are no markers or arm location information for *H20*. It has not been widely used for wheat breeding and is difficult to compare without marker or location information. *H21* was identified from ‘Chaupon’ rye on 2RL [65]. Translocation line T2BS.2RL was used for introgression to wheat. However, *H21* also has not been widely used due to the linkage drag [66]. Therefore, it is likely that the QTL found on 2B could be a newly documented *H* gene location.

**Fig 6:**
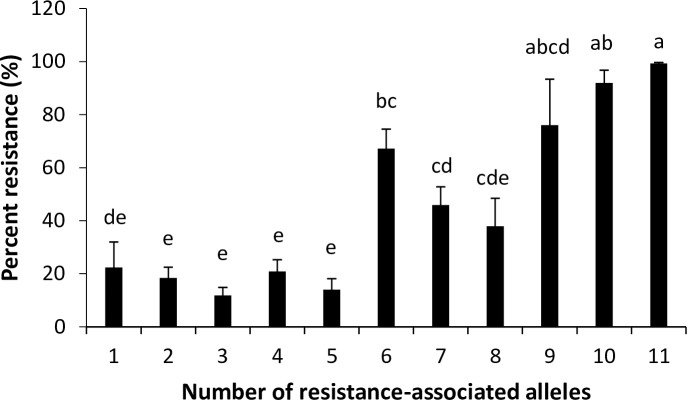
Number of favorable alleles for 6BS haplotypes and mean percentage of resistance to infection by UI Hessian fly population. Bars with the same letter are not significantly different at *P* < 0.05 (Tukey-Kramer HSD). Each value is the mean ± standard error.

A total of three loci associated with HF resistance were detected on 6BS. Genotypes were resistant to the UI HF population when more than 9 marker alleles associated with resistance were present with phenotypic means of 76.0% and greater (Fig 6). Thirty-five genotypes had eleven resistance-associated marker alleles on 6BS and the majority of them were from Washington State University. Twenty-one genotypes had ten resistance-associated marker alleles and 16 of them did not have the favorable allele of IWA7841 suggesting having favorable allele of SNPs on *QHf*.*pnw*.*6B*.*1* and *QHf*.*pnw*.*6B*.*2* is strongly correlated with the resistant phenotype. IWA921 positioned in *QHf*.*pnw*.*6B*.*2* was identified as one of the flanking markers for *H34* [67]. Other flanking markers reported for *H34* were either not significant (*P* < 0.001) or were excluded from the analysis since they did not meet the filtering criteria. The QTL tagging SNP for *QHf*.*pnw*.*6B*.*2*, IWA6803, explained 14.3% variation (R^2^) and met Bonferroni correlation criteria (*P* < 0.1) and may be closely linked to *H34*. Elite experimental line WA008076, one of the genotypes with confirmed strong resistance to both HF populations, has ten favorable alleles and has been used to validate and fine-map resistance to Hessian fly on 6BS in a doubled haploid population (unpublished data).

*QHf*.*pnw*.*6A*.*2* on the distal end of 6AS around 6.2 cM conferred a large effect. However, QTL-tagged SNP (IWA6803) in this locus conferring the large effect also had a locus in the distal end of 6BS, *QHf*.*pnw*.*6B*.*2*. These SNPs were in LD to each other, moreover other SNPs in the loci which did not map in two chromosomes were also in LD with IWA6803. One of QTL-tagging SNPs, IWA3982, was also detected in two loci, *QHf*.*pnw*.*2B* and *QHf*.*pnw*.*6B*.*1*. Cavanagh et al. [15] reported that there are 322 SNPs that were mapped for two loci. There are no published reports of *H* genes on 6AS. Therefore, it is possible that detection of the *QHf*.*pnw*.*6A*.*1* and *QHf*.*pnw*.*6A*.*2* are an artifact of issues with homoeologous allele genotyping of loci on 6BS or there are resistance loci which remained uncharacterized. Also, it could be the same for *QHf*.*pnw*.*2B*. Hessian fly resistance related loci detected on 4BS is also in a location not previously reported with designated *H* genes. These newly detected loci will require further validation via genetic analysis. Given the dynamic nature of HF population evolution, additional loci would aid in diversifying the genetic basis of HF resistance.

### *Septoria tritici* blotch resistance screening

Overall, STB disease pressure was adequate as seen in high disease ranges regardless of location (Table 5). Disease severity in 2013 showed lower means and smaller range which suggest that disease arrived late compare to the 2014 ratings even though data were taken at the same Zadoks stage. Mean and range of disease ratings were much higher in 2014. There were 63 genotypes with 100% disease severity in both locations in 2014. Correlation coefficients for disease severity were significant across the locations and years (Table 6). Analysis of variance for STB phenotypes in three environments (S5 Table) showed that the variance component for genotype (*σ*^2^_*G*_) and GxE (*σ*^2^_*GE*_) were highly significant (*P< 0*.*001*); variance for environment (*σ*^2^_*E*_) was not significantly different. Heritability of STB resistance was 0.2, revealing substantial variation possibly due to variable STB races and environmental variation combined with complex genetics of resistance.

**Table 5:** Statistical parameters for disease severity (%) response to *Septoria tritici* blotch in Holeta (2013 and 2014) and Bekoji (2014), Ethiopia.

	HT13	HT14	BJ14
**Mean**	21.5	92.3	79.8
**SE**	1.4	1.0	1.5
**Range**	2.5–69.1	17.3–100	22.2–100

HT—Holeta, Ethiopia; BJ—Bekoji, Ethiopia followed by year, SE–standard error.

**Table 6:** Pearson correlation coefficients for disease severity (%) and plant height to *Septoria tritici* blotch in Holeta (2013 and 2014) and Bekoji (2014), Ethiopia.

	HT13	HT14	BJ14	PHT
**HT13**	1.0			
**HT14**	0.28**	1.0		
**BJ14**	0.53***	0.47***	1.0	
**PHT**	0.20*	0.03^ns^	0.04^ns^	1.0

HT—Holeta, Ethiopia; BJ—Bekoji, Ethiopia followed by year, PHT—plant height.

^ns^, *, **, ***P value nonsignificant, significant at Pearsons correlation *P <* 0.05, *P* < 0.01 or *P* ≤ 0.001, respectively.

STB infection can be influenced by plant architecture such as height since spores are often splash-dispersed [68–70]. Disease severity rated in HT13 was significantly, but not strongly correlated with plant height (Table 6). To account for the PHT effect, PHT data was considered as a covariate for HT13 GWAS analysis. GWAS results with or without PHT as covariate revealed that results were no different from each other. Therefore, all the analysis for STB were done without including PHT as covariate.

The overall GWAS result which combined data across location-years yielded no MTAs strong enough to meet Bonferroni threshold (*P* < 0.1) or FDR (*P* < 0.1). It is possible that we did not see strong marker-trait association between the subpanel and STB since the panel does not contain effective R genes for Ethiopian STB races. Eight loci on six chromosomes, 2A, 2B, 3B, 6A, 6B, 7A and 7B, were detected and multiple loci were found on chromosomes 3B and 7B (Table 7). No SNPs or QTL were found commonly among the environments.

**Table 7:** Chromosome location and *R*^*2*^ values of significantly associated SNP markers with *Septoria tritici* blotch resistance at Holeta (2013 and 2014) and Bekoji (2014), Ethiopia, from the Northwestern elite spring wheat sub-population panel.

SNP	Chr.^a^	Position^a^	HT13	HT14	BJ14	*Stb*/QTL
IWA8491	2A	88.3	0.2^b^	1.2	6.3	*MQTL4*
IWA6263	2B	2.2	7.1	1.6	3.8	*QTL3*
IWA4653	3B	94.2	0.0	5.6	0.2	*QStb*.*lsa_af-3B*, *MQTL14*
IWA3901		123	0.0	5.7	1.5	*MQTL14 QStb*.*cim-3BL*
IWA3207	6A	76.9	1.6	0.2	6.0	*Stb15*
IWA8011	6B	56	1.8	6.8	2.1	*Qstb*.*psr-6B-1*, *Qstb*.*6B*, *QStb*.*riso-6B*.*2*
IWA814	7B	52.5	5.7	1.2	1.8	*QStb*.*riso-7B*
IWA3513		139.2	2.2	5.7	1.5	*QStb*.*ipk-7B*

Chr.–chromosome, HT–Holeta, BJ–Bekoji, followed by year.

^a^Chromosome location and position was described in [15].

^b^Phenotype variation explained by the marker in percent (*R*^*2*^)

**Fig 7:**
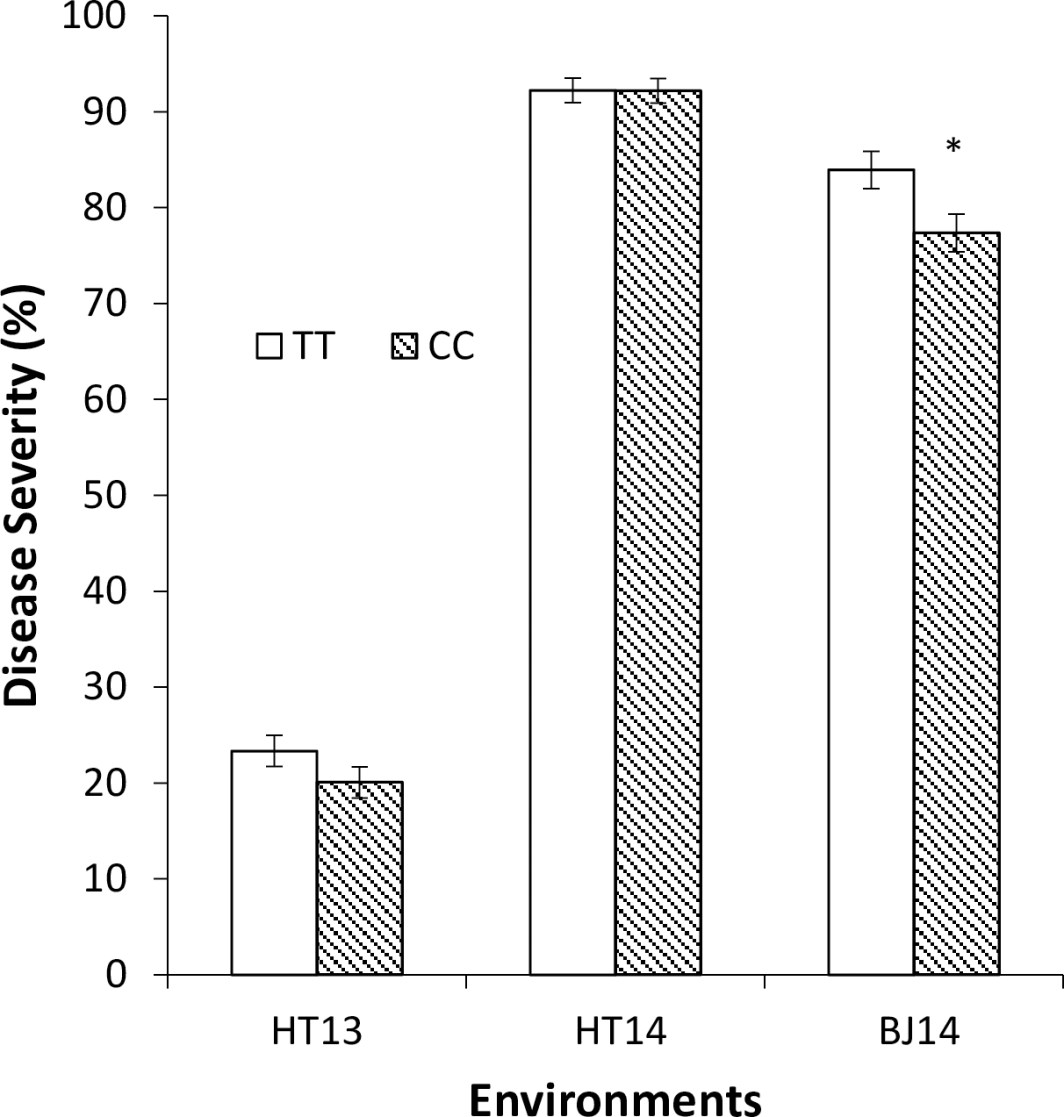
Mean *Septoria tritici* blotch disease severity by susceptible (TT) and resistant (CC) alleles for IWA3207 in 3 environments. HT13 –Holeta 2013; HT14 –Holeta 2014; BJ14 –Bekoji 2014. Each value is the mean ± standard error. Asterisk indicates that TT and CC alleles within an environment were significantly different from each other (ANOVA, *P* < 0.05).

The QTL locations were compared with the 20 known *Stb* gene and QTL locations. Three designated *Stb* genes, *Stb8*, *Stb13*, and *Stb15* [71–73], are positioned on the same chromosome arms as QTL detected by our GWAS. The position reported for *Stb13* on 7BL on the consensus map by Maccafferri et al. [22] and the *Stb* concensus map by Brown et al. [13] were distant from the MTAs we detected. However, the other locus on 7BL was in proximity to the *Stb8*. *Stb8* was identified from synthetic hexaploid wheat, W7984, which showed adult stage resistance. This line was bred by CIMMYT (International Maize and Wheat Improvement Center) by crossing durum wheat, Altar 84, and *Aegilops tauschii*. A QTL found on chromosome 6AS, tagged by IWA3207, fell within *Stb15* region. Interestingly, *Stb15* was originally found based on seedling stage resistance to an Ethiopian isolate, IPO88004 [72], and is commonly present in current European wheat [13,74]. In this panel, 130 genotypes (66%) had the resistance-associated allele for IWA3207 (S1 File). Genotypes with the IWA3207 resistance-associated allele had significantly lower mean disease severity percentage in BJ14 (Fig 7). The effect of this locus explained 6% of the variation in STB resistance in BJ14. Except for the three above, all other QTL locations coincided with previously reported STB QTL locations.

## Conclusion

Markers detected by GWAS provide higher resolution than linkage mapping [75]. Also, markers developed by linkage mapping are often not transferrable to widely diverse sets of genotypes [76]. Our GWAS of multiple pest resistances in a spring wheat panel of Northwestern United States germplasm identified many loci associated with economically important traits. As we have demonstrated by screening for multiple pest resistances, once a panel is genotyped, additional traits can be characterized to provide a comprehensive genetic architecture for the panel. QTL position identified in this study correspond to previously documented resistance loci, in some cases identical SNP markers were identified for the same traits. Detecting QTL previously reported in other GWAS or bi-parental mapping studies provides robust validation of our results. Furthermore, analysis of this panel also resulted in the detection of resistant loci not previously reported.

One MTA detected for stripe rust resistance was validated by an RIL population included in this study. The importance of having a bi-parental mapping population to verify the GWAS result is confirmed as was seen by other GWAS studies in wheat and maize [18,77].

Tagging SNPs discovered in this study can be converted to KASP or similar probes for marker assisted selection, or be used as a starting point for higher-resolution mapping and diagnostic marker development. Also, incorporating locus-specific prior information for genomic selection (GS) analysis significantly increase the accuracy of analysis [78,79]. Therefore, the tagging SNPs can be included into a protocol like the spiked genotyping-by-sequence method (sGBS) for GS which allows whole-genome profiling and genotyping of multiple single-target loci simultaneously [80].

This study demonstrated that once a diverse panel adapted to a specific region is formed and genotyped with high-density SNP markers, it can then be screened with multiple traits of interest and informative loci can be found. For example, we were able to identify 55 loci for three traits. Valuable information on potential coupling or repulsion linkage among pest resistance traits, pleiotropic resistance loci, and linkage relationships with other key agronomic and end-use quality traits can be further characterized and exploited by wheat breeders. These findings will help to direct future use of genotypic data in selection for multiple pest resistances, and provide a reference for future research on specific loci.

## Supporting information

S1 File The Northwestern elite spring wheat panel genotype information. (XLSX)

S2 File 9K SNP data for the elite Northwestern spring wheat panel (n = 395). SNPs were filtered for 10% MAF (6,086 SNPs).
(XLSX)

S3 File All phenotypic data used for GWAS in this study. (XLSX)

S1 Table Chromosome locations and *P* values of significantly associated SNP markers with seedling infection type. (DOCX)

S2 Table Chromosome locations and *P* values of significantly associated SNP markers with adult infection type. (DOCX)

S3 Table Chromosome locations and *P* values of significantly associated SNP markers with adult disease severity. (DOCX)

S4 Table Chromosome locations and *R*^*2*^ values of significantly associated SNP markers at unique stage(s) and disease phenotype. (DOCX)

S5 Table Analysis of variance (ANOVA) for disease severity (%) response to *Septoria tritici* blotch (STB) in Holeta and Bekoji, Ethiopia. (DOCX)
