# Supplementary material for: Genome-wide associations for multiple pest resistances in a Northwestern United States elite spring wheat panel
Source: PLoS One. 2018 Feb 7;13(2):e0191305. doi: 10.1371/journal.pone.0191305 (PMC5802848; doi:10.1371/journal.pone.0191305)
Supplement: S1 Table — (DOCX) [file pone.0191305.s004.docx]

**S1 Table. Chromosome location and *P* values of significantly associated SNP markers with seedling infection type.**

| **Marker** | **Chr^.^** | **Pos^a^.** | **SP11** | **MV12** | **MV13** |
| --- | --- | --- | --- | --- | --- |
| IWA63 | 1B | 18.1 | 2.40E-04 | 9.4E-05^*^ | 2.3E-11^*^ |
| IWA2583 |  | 18.4 | 2.60E-03 | 1.3E-04^*^ | 1.7E-09^***^ |
| IWA8557 |  | 18.7 | 1.40E-01 | 6.90E-03 | 1.6E-04^*^ |
| IWA1191 |  | 23.7 | 4.60E-02 | 1.2E-05^**^ | 1.3E-08^**^ |
| IWA2577 |  | 23.7 | 1.20E-01 | 2.00E-02 | 6.4E-05^*^ |
| IWA7117 |  | 23.7 | 1.10E-01 | 7.80E-03 | 1.7E-04^*^ |
| IWA1566 |  | 28.1 | 1.50E-01 | 2.0E-06^**^ | 5.0E-10^**^ |
| IWA2150 |  | 28.2 | 6.00E-02 | 2.3E-08^**^ | 3.0E-07^**^ |
| IWA2561 |  | 28.1 | 5.60E-02 | 3.5E-06^**^ | 3.9E-09^**^ |
| IWA2881 |  | 28.1 | 9.40E-02 | 6.6E-06^**^ | 4.5E-10^**^ |
| IWA4093 |  | 28.1 | 3.90E-01 | 2.3E-06^**^ | 1.8E-07^**^ |
| IWA6610 |  | 28.1 | 3.30E-01 | 2.7E-06^**^ | 1.2E-07^**^ |
| IWA6611 |  | 28.1 | 1.70E-01 | 8.2E-06^**^ | 1.3E-07^**^ |
| IWA8275 |  | 28.2 | 7.60E-02 | 8.4E-09^**^ | 2.8E-07^**^ |
| IWA3816 |  | 30.5 | 1.20E-01 | 2.20E-03 | 1.6E-04^*^ |
| IWA3620 |  | 33.4 | 5.10E-01 | 1.40E-01 | 2.7E-04^*^ |
| IWA4975 |  | 33.4 | 3.00E-01 | 1.40E-01 | 2.7E-04^*^ |
| IWA7737 |  | 33.4 | 3.10E-01 | 1.40E-01 | 1.0E-04^*^ |
| IWA4504 |  | 30.5 | 4.00E-02 | 2.10E-02 | 9.10E-04 |
| IWA6449 |  | 40.4 | 7.40E-02 | 3.10E-02 | 9.60E-04 |
| IWA6450 |  | 40.4 | 3.50E-02 | 1.60E-02 | 5.80E-04 |
| IWA7638 | 2A | 243.8 | 2.00E-01 | 2.00E-02 | 6.10E-04 |
| IWA8599 | 2B | 70.2 | 5.10E-04 | 5.20E-03 | 1.90E-02 |
| IWA2379 |  | 195.8 | 1.30E-04 | 2.60E-01 | 4.50E-01 |
| IWA2701 |  | 195.8 | 1.5E-05^**^ | 1.50E-01 | 2.20E-01 |
| IWA2702 |  | 195.8 | 4.3E-05^*^ | 2.80E-01 | 5.00E-02 |
| IWA2678 |  | 196.8 | 2.40E-04 | 1.50E-01 | 1.40E-01 |
| IWA2873 |  | 202.2 | 1.5E-05^**^ | 4.80E-01 | 3.90E-01 |
| IWA3176 |  | 202 | 2.6E-05^*^ | 8.90E-01 | 5.80E-01 |
| IWA7640 |  | 210.2 | 7.20E-04 | 4.90E-01 | 4.10E-02 |
| IWA7009 | 5A | 166.4 | 5.30E-01 | 2.80E-04 | 3.80E-01 |
| IWA6902 | 5B | 205 | 3.4E-05^*^ | 1.10E-01 | 5.40E-03 |

^a^SNP chromosome and position information was derived from Cavanagh et al., 2013

^*,**^*P* value significant at FDR 0.1 or Bonferroni 0.1, respectively

**Reference:** Cavanagh C, Chao S, Wang S, Huang BE, Stephen S. Genome-wide comparative diversity uncovers multiple targets of selection for improvement in hexaploid wheat landraces and cultivars. Proc Natl Acad Sci USA. 2013;110: 8057–8062.
