# Supplementary material for: Genome-wide associations for multiple pest resistances in a Northwestern United States elite spring wheat panel
Source: PLoS One. 2018 Feb 7;13(2):e0191305. doi: 10.1371/journal.pone.0191305 (PMC5802848; doi:10.1371/journal.pone.0191305)
Supplement: S2 Table — (DOCX) [file pone.0191305.s005.docx]

**S2 Table. Chromosome location and *P* values of significantly associated SNP markers with adult infection type.**

| **Marker** | **Chr^a^.** | **Pos^a^.** | **SP11** | **SP12** | **SP13** | **MV12** | **MV13** | **WL12** | **WL13** |
| --- | --- | --- | --- | --- | --- | --- | --- | --- | --- |
| IWA63 | 1B | 18.1 | 2.30E-01 | 6.50E-05 | 1.1E-07^**^ | 4.8E-07^**^ | 3.1E-06^**^ | 3.3E-06^**^ | 2.9E-07^**^ |
| IWA2583 |  | 18.4 | 2.40E-01 | 1.20E-04 | 3.4E-07^**^ | 2.9E-07^**^ | 4.5E-07^**^ | 1.6E-06^**^ | 4.7E-08^**^ |
| IWA1191 |  | 23.7 | 2.30E-01 | 2.10E-05 | 3.0E-06^**^ | 6.0E-05^*^ | 6.60E-04 | 1.8E-06^**^ | 7.8E-06^**^ |
| IWA2577 |  | 23.7 | 5.90E-02 | 1.40E-03 | 1.4E-04^*^ | 8.20E-03 | 4.80E-03 | 1.80E-04 | 1.80E-03 |
| IWA7117 |  | 23.7 | 7.40E-02 | 1.30E-03 | 6.40E-04 | 4.30E-02 | 2.20E-03 | 4.0E-05^*^ | 1.00E-02 |
| IWA7578 |  | 26.3 | 9.40E-02 | 1.30E-02 | 3.30E-02 | 3.70E-04 | 2.10E-02 | 3.10E-02 | 1.60E-02 |
| IWA2881 |  | 28.1 | 2.90E-01 | 6.00E-05 | 5.0E-06^**^ | 2.0E-04^*^ | 3.6E-05^*^ | 3.20E-04 | 3.0E-07^**^ |
| IWA2561 |  | 28.1 | 1.00E-01 | 8.60E-05 | 5.4E-07^**^ | 8.4E-06^**^ | 5.1E-06^**^ | 8.40E-04 | 2.0E-06^**^ |
| IWA6611 |  | 28.1 | 1.50E-01 | 1.00E-03 | 1.4E-04^*^ | 7.2E-05^*^ | 2.30E-04 | 1.70E-03 | 3.8E-05^*^ |
| IWA1566 |  | 28.1 | 5.00E-01 | 1.10E-03 | 1.70E-04 | 3.40E-04 | 4.2E-05^*^ | 1.30E-02 | 3.1E-05^*^ |
| IWA4093 |  | 28.1 | 3.80E-01 | 1.40E-03 | 5.10E-04 | 2.90E-04 | 3.60E-04 | 1.40E-02 | 4.1E-05^*^ |
| IWA6610 |  | 28.1 | 4.10E-01 | 2.20E-03 | 1.10E-03 | 5.70E-04 | 5.90E-04 | 1.10E-02 | 8.7E-0^*^ |
| IWA2150 |  | 28.2 | 2.10E-01 | 6.40E-04 | 9.9E-06^**^ | 2.90E-04 | 2.4E-05^*^ | 2.40E-03 | 1.6E-06^**^ |
| IWA8275 |  | 28.2 | 2.20E-01 | 7.70E-04 | 1.1E-05^**^ | 8.40E-04 | 4.5E-05^*^ | 1.20E-03 | 5.7E-07^**^ |
| IWA3816 |  | 30.5 | 3.10E-01 | 1.10E-03 | 2.50E-04 | 2.60E-02 | 4.10E-04 | 3.30E-02 | 1.4E-04^*^ |
| IWA6450 |  | 40.4 | 1.50E-01 | 3.50E-03 | 4.00E-03 | 3.10E-04 | 1.90E-02 | 7.70E-04 | 6.20E-04 |
| IWA6449 |  | 40.4 | 2.30E-01 | 1.10E-02 | 9.30E-03 | 4.90E-04 | 1.50E-02 | 8.80E-04 | 8.30E-04 |
| IWA4504 |  | 40.4 | 1.30E-01 | 1.70E-02 | 1.00E-02 | 2.70E-04 | 3.40E-02 | 2.00E-03 | 1.70E-03 |
| IWA2504 |  | 47.5 | 1.3E-05^**^ | 1.80E-01 | 1.60E-02 | 5.70E-01 | 6.00E-03 | 1.30E-02 | 4.70E-02 |
| IWA3348 |  | 47.5 | 3.50E-05 | 8.60E-02 | 1.60E-02 | 5.30E-01 | 5.80E-03 | 3.10E-02 | 8.50E-03 |
| IWA7466 |  | 47.5 | 6.90E-04 | 2.80E-01 | 4.80E-01 | 7.70E-01 | 3.40E-02 | 3.30E-01 | 3.90E-01 |
| IWA5418 |  | 47.5 | 7.30E-04 | 3.20E-01 | 5.30E-01 | 7.90E-01 | 6.00E-02 | 4.00E-01 | 4.20E-01 |
| IWA7017 |  | 56.7 | 7.90E-04 | 2.40E-02 | 2.70E-03 | 4.10E-01 | 1.70E-02 | 1.30E-01 | 3.00E-02 |
| IWA7876 | 2A | 186.6 | 1.10E-01 | 1.30E-01 | 1.70E-01 | 7.80E-02 | 8.50E-01 | 9.90E-04 | 7.20E-02 |
| IWA7638 |  | 243.8 | 1.30E-02 | 2.50E-03 | 3.60E-03 | 8.50E-04 | 6.40E-03 | 1.40E-02 | 4.50E-03 |
| IWA4866 | 2B | 194.8 | 1.70E-04 | 3.60E-02 | 4.30E-02 | 1.40E-01 | 5.00E-01 | 5.30E-01 | 1.50E-01 |
| IWA2179 |  | 194.8 | 2.80E-04 | 1.00E-02 | 2.50E-02 | 1.20E-01 | 3.90E-01 | 2.50E-01 | 6.40E-02 |
| IWA2702 |  | 195.8 | 8.00E-04 | 2.40E-03 | 7.30E-03 | 2.00E-02 | 2.00E-01 | 4.90E-02 | 1.40E-02 |
| IWA3148 |  | 200.1 | 1.70E-03 | 2.20E-04 | 4.20E-03 | 3.20E-02 | 2.00E-02 | 3.00E-02 | 5.30E-03 |
| IWA3938 |  | 211.1 | 6.10E-03 | 3.20E-02 | 3.80E-02 | 1.90E-03 | 7.40E-04 | 2.50E-04 | 3.70E-02 |
| IWA8266 |  | 211.8 | 4.50E-02 | 3.90E-04 | 1.50E-01 | 2.90E-02 | 5.80E-02 | 9.40E-03 | 5.80E-03 |
| IWA4796 | 3B | 1.9 | 1.60E-04 | 1.10E-02 | 4.40E-02 | 2.10E-03 | 9.10E-02 | 9.50E-02 | 1.20E-01 |
| IWA5203 |  | 3.7 | 5.10E-05 | 1.00E-02 | 7.60E-02 | 1.80E-01 | 1.20E-01 | 5.20E-02 | 2.00E-01 |
| IWA5202 |  | 3.9 | 1.10E-04 | 2.90E-02 | 9.60E-02 | 1.30E-01 | 2.10E-01 | 4.00E-02 | 2.60E-01 |
| IWA3103 |  | 4.2 | 7.00E-05 | 1.10E-02 | 5.80E-02 | 7.70E-02 | 1.60E-01 | 3.20E-02 | 2.20E-01 |
| IWA5201 |  | 4.2 | 8.60E-04 | 1.30E-01 | 4.80E-01 | 2.40E-01 | 3.10E-02 | 3.20E-01 | 2.30E-01 |
| IWA2470 | 4B | 124.3 | 2.00E-04 | 1.00E-02 | 7.10E-02 | 1.40E-01 | 1.60E-01 | 4.10E-02 | 1.20E-01 |
| IWA2469 |  | 124.3 | 2.40E-04 | 5.00E-02 | 4.50E-02 | 8.10E-02 | 2.20E-01 | 5.80E-02 | 7.40E-02 |
| IWA2087 |  | 124.3 | 2.90E-04 | 8.10E-02 | 3.60E-01 | 1.90E-01 | 4.40E-01 | 3.30E-02 | 2.70E-01 |
| IWA1798 |  | 124.3 | 4.00E-04 | 4.90E-02 | 1.60E-01 | 1.50E-01 | 3.00E-01 | 3.80E-02 | 9.10E-02 |
| IWA4615 |  | 124.5 | 2.60E-04 | 1.60E-02 | 5.80E-02 | 6.10E-02 | 2.40E-01 | 3.70E-02 | 5.70E-02 |
| IWA4618 |  | 124.5 | 5.00E-04 | 3.10E-02 | 1.80E-01 | 2.40E-01 | 1.40E-01 | 3.70E-02 | 6.80E-02 |
| IWA7299 |  | 124.9 | 1.90E-04 | 5.40E-02 | 1.90E-01 | 1.70E-01 | 1.90E-01 | 2.80E-02 | 1.30E-01 |
| IWA2146 | 5A | 12.9 | 3.10E-01 | 4.60E-01 | 5.40E-01 | 7.60E-01 | 6.90E-04 | 9.20E-02 | 3.40E-02 |
| IWA2145 |  | 12.9 | 4.60E-01 | 2.90E-01 | 3.20E-01 | 4.30E-01 | 9.60E-04 | 1.40E-01 | 3.40E-02 |
| IWA6405 |  | 36.8 | 5.00E-01 | 1.00E-02 | 6.20E-02 | 1.00E-01 | 5.60E-01 | 5.20E-04 | 7.90E-03 |
| IWA1280 |  | 54.2 | 8.30E-05 | 5.30E-03 | 2.60E-03 | 4.70E-02 | 1.30E-04 | 8.90E-02 | 2.00E-03 |
| IWA2282 |  | 162.9 | 6.00E-01 | 1.10E-02 | 2.10E-01 | 5.20E-04 | 1.90E-01 | 9.60E-02 | 1.00E-01 |
| IWA5950 | 5B | 152.4 | 5.90E-02 | 1.40E-01 | 9.30E-04 | 3.50E-01 | 3.00E-02 | 2.10E-03 | 5.20E-04 |
| IWA2255 |  | 152.8 | 1.90E-02 | 6.20E-02 | 9.70E-04 | 2.80E-02 | 8.20E-03 | 4.90E-03 | 1.70E-02 |
| IWA7989 |  | 156.7 | 1.60E-02 | 1.10E-01 | 1.60E-02 | 4.90E-02 | 3.60E-03 | 9.30E-05 | 4.30E-02 |
| IWA1427 | 5D | 0 | 3.20E-03 | 2.80E-04 | 9.70E-03 | 2.90E-02 | 9.30E-02 | 2.40E-02 | 4.50E-03 |
| IWA1428 |  | 0 | 3.50E-03 | 3.00E-04 | 1.10E-02 | 2.10E-02 | 1.20E-01 | 3.50E-02 | 8.20E-03 |
| IWA1429 |  | 0 | 5.20E-03 | 7.40E-04 | 8.90E-03 | 3.60E-02 | 5.60E-02 | 3.40E-02 | 5.10E-03 |
| IWA1493 | 6B | 0.6 | 2.60E-04 | 3.40E-01 | 1.70E-01 | 8.10E-01 | 6.60E-01 | 6.40E-01 | 1.90E-01 |
| IWA1531 |  | 96.3 | 9.50E-04 | 1.60E-02 | 4.70E-02 | 4.40E-01 | 1.70E-01 | 7.60E-02 | 4.30E-01 |
| IWA6507 | 7A | 40.6 | 3.10E-02 | 1.00E-01 | 1.30E-01 | 1.3E-04^*^ | 2.70E-02 | 9.60E-01 | 2.40E-02 |
| IWA5390 | 7B | 19.8 | 1.30E-01 | 1.70E-01 | 1.10E-01 | 2.60E-01 | 2.60E-04 | 5.10E-02 | 2.00E-02 |
| IWA4857 |  | 100.4 | 1.50E-01 | 7.30E-02 | 3.40E-03 | 1.50E-02 | 4.70E-03 | 8.30E-02 | 9.30E-04 |

^a^SNP chromosome, and position information was derived from Cavanagh et al., 2013

^*,**^*P* value significant at FDR 0.1 or Bonferroni 0.1, respectively

**Reference:** Cavanagh C, Chao S, Wang S, Huang BE, Stephen S. Genome-wide comparative diversity uncovers multiple targets of selection for improvement in hexaploid wheat landraces and cultivars. Proc Natl Acad Sci USA. 2013;110: 8057–8062.
