# Supplementary material for: Genome-wide associations for multiple pest resistances in a Northwestern United States elite spring wheat panel
Source: PLoS One. 2018 Feb 7;13(2):e0191305. doi: 10.1371/journal.pone.0191305 (PMC5802848; doi:10.1371/journal.pone.0191305)
Supplement: S3 Table — (DOCX) [file pone.0191305.s006.docx]

**S3 Table. Chromosome location and *P* values of significantly associated SNP markers with adult disease severity**.

| **Marker** | **Chr^a^.** | **Pos^a^.** | **SP11** | **SP12** | **SP13** | **MV12** | **MV13** | **WL12** | **WL13** |
| --- | --- | --- | --- | --- | --- | --- | --- | --- | --- |
| IWA63 | 1B | 18.1 | 8.70E-05 | 9.60E-04 | 3.2E-05^*^ | 6.8E-06^**^ | 2.20E-04 | 9.6E-07^**^ | 1.00E-04 |
| IWA2583 |  | 18.4 | 9.60E-05 | 4.80E-04 | 2.8E-05^*^ | 3.4E-06^**^ | 1.10E-04 | 1.8E-07^**^ | 1.0E-05^**^ |
| IWA6889 |  | 21.7 | 5.20E-02 | 1.30E-02 | 1.50E-02 | 6.20E-04 | 2.20E-02 | 1.80E-02 | 1.80E-02 |
| IWA4678 |  | 22.5 | 8.30E-02 | 2.00E-01 | 3.30E-02 | 9.60E-04 | 4.80E-02 | 1.20E-02 | 4.60E-03 |
| IWA1191 |  | 23.7 | 3.60E-04 | 8.90E-03 | 2.10E-04 | 5.80E-03 | 1.40E-02 | 1.90E-04 | 3.70E-05 |
| IWA7117 |  | 23.7 | 4.20E-02 | 6.40E-04 | 1.60E-02 | 3.10E-01 | 1.10E-01 | 2.50E-03 | 2.00E-02 |
| IWA1566 |  | 28.1 | 7.70E-03 | 1.80E-02 | 7.10E-03 | 1.20E-02 | 3.90E-02 | 4.5E-05^*^ | 1.80E-03 |
| IWA2150 |  | 28.2 | 1.60E-02 | 4.50E-03 | 1.20E-03 | 8.20E-04 | 1.90E-02 | 2.60E-04 | 5.30E-04 |
| IWA2561 |  | 28.1 | 8.30E-03 | 7.00E-04 | 5.80E-04 | 6.00E-04 | 5.60E-03 | 1.4E-06^**^ | 4.40E-04 |
| IWA2881 |  | 28.1 | 1.60E-02 | 4.30E-04 | 1.60E-03 | 4.50E-03 | 3.30E-02 | 2.6E-05^*^ | 1.90E-04 |
| IWA4093 |  | 28.1 | 2.20E-02 | 9.70E-03 | 2.90E-03 | 2.00E-02 | 7.80E-02 | 1.60E-04 | 3.30E-03 |
| IWA6610 |  | 28.1 | 3.10E-02 | 2.60E-02 | 3.20E-03 | 1.70E-02 | 8.90E-02 | 2.00E-04 | 2.90E-03 |
| IWA6611 |  | 28.1 | 2.50E-03 | 6.10E-03 | 6.10E-04 | 7.10E-03 | 7.60E-02 | 5.2E-05^*^ | 9.20E-04 |
| IWA8275 |  | 28.2 | 1.10E-02 | 7.20E-03 | 9.90E-04 | 4.90E-04 | 1.50E-02 | 1.40E-04 | 2.30E-04 |
| IWA3816 |  | 30.5 | 2.40E-02 | 2.00E-02 | 1.40E-01 | 3.40E-02 | 6.40E-02 | 6.40E-04 | 2.50E-03 |
| IWA6450 |  | 40.4 | 1.90E-02 | 1.20E-02 | 8.50E-02 | 1.20E-02 | 4.40E-03 | 1.60E-03 | 6.90E-04 |
| IWA3143 | 2A | 5.9 | 2.70E-02 | 2.20E-02 | 3.20E-03 | 9.80E-04 | 2.30E-03 | 9.40E-02 | 3.60E-03 |
| IWA2179 | 2B | 194.8 | 3.20E-04 | 1.20E-03 | 4.60E-02 | 1.30E-02 | 1.90E-02 | 5.40E-02 | 1.40E-01 |
| IWA2379 |  | 30.5 | 2.7E-05^*^ | 2.60E-03 | 2.40E-02 | 1.00E-01 | 1.90E-01 | 1.60E-03 | 1.30E-02 |
| IWA2701 |  | 195.8 | 6.4E-06^**^ | 1.40E-03 | 2.10E-02 | 2.50E-02 | 4.00E-02 | 1.60E-03 | 1.20E-02 |
| IWA2702 |  | 195.8 | 2.9E-05^*^ | 2.80E-03 | 2.20E-02 | 1.20E-02 | 1.30E-02 | 6.00E-04 | 2.80E-03 |
| IWA2678 |  | 196.8 | 8.00E-04 | 3.90E-03 | 3.10E-02 | 1.40E-02 | 5.90E-02 | 1.20E-03 | 2.80E-02 |
| IWA4098 |  | 197.7 | 1.30E-04 | 3.70E-03 | 8.50E-02 | 2.30E-02 | 1.60E-02 | 3.80E-04 | 1.10E-01 |
| IWA2676 |  | 200.1 | 5.10E-04 | 2.60E-03 | 2.90E-02 | 4.80E-03 | 1.60E-02 | 2.20E-03 | 2.00E-02 |
| IWA4097 |  | 200.5 | 2.70E-04 | 1.70E-03 | 1.30E-01 | 5.10E-02 | 1.20E-02 | 5.40E-03 | 1.60E-01 |
| IWA3938 |  | 211.1 | 4.60E-03 | 6.70E-04 | 4.30E-03 | 2.60E-03 | 1.00E-02 | 9.80E-03 | 1.10E-02 |
| IWA8266 |  | 211.8 | 6.70E-04 | 2.70E-04 | 1.70E-02 | 1.10E-01 | 4.00E-03 | 9.00E-04 | 5.00E-03 |
| IWA7113 |  | 219.9 | 4.80E-01 | 2.70E-01 | 8.60E-04 | 5.40E-02 | 1.00E-01 | 1.30E-01 | 2.60E-01 |
| IWA3594 |  | 249.5 | 9.20E-04 | 2.70E-01 | 2.20E-01 | 2.40E-01 | 1.70E-01 | 2.30E-01 | 1.90E-01 |
| IWA4796 | 3B | 1.9 | 2.80E-02 | 6.00E-03 | 1.90E-01 | 1.30E-04 | 2.20E-02 | 1.60E-01 | 2.30E-01 |
| IWA1617 |  | 75.7 | 5.70E-01 | 1.40E-01 | 3.30E-01 | 6.40E-04 | 1.80E-01 | 1.60E-02 | 4.30E-02 |
| IWA8058 |  | 165.8 | 6.00E-01 | 2.90E-01 | 7.90E-03 | 2.50E-02 | 7.50E-04 | 5.60E-02 | 2.00E-02 |
| IWA1992 | 4A | 44 | 1.20E-01 | 6.30E-04 | 5.70E-01 | 1.50E-02 | 1.60E-01 | 9.70E-02 | 7.00E-01 |
| IWA2170 |  | 167.3 | 3.10E-01 | 9.60E-04 | 5.70E-02 | 1.50E-01 | 9.40E-02 | 9.10E-03 | 1.20E-02 |
| IWA1062 | 5A | 31.4 | 7.70E-02 | 2.80E-01 | 1.50E-01 | 3.90E-02 | 3.70E-02 | 2.40E-01 | 8.20E-04 |
| IWA3989 |  | 112 | 3.60E-02 | 6.30E-01 | 1.20E-01 | 3.10E-02 | 3.00E-01 | 5.00E-02 | 3.70E-04 |
| IWA3990 |  | 112 | 3.20E-02 | 5.10E-01 | 1.30E-01 | 2.90E-02 | 2.70E-01 | 6.50E-02 | 6.40E-04 |
| IWA6988 |  | 190.4 | 6.50E-02 | 9.80E-01 | 7.30E-02 | 3.40E-02 | 1.40E-02 | 1.40E-02 | 2.20E-04 |
| IWA7815 | 5B | 121.8 | 3.90E-02 | 3.80E-02 | 7.80E-05 | 1.20E-03 | 1.30E-02 | 3.60E-02 | 5.70E-03 |
| IWA7989 |  | 156.7 | 5.50E-02 | 2.00E-01 | 1.20E-02 | 4.00E-01 | 6.80E-04 | 2.30E-02 | 6.30E-01 |
| IWA584 |  | 172.5 | 9.50E-04 | 9.20E-01 | 1.90E-02 | 6.80E-01 | 1.30E-02 | 7.50E-02 | 3.60E-02 |
| IWA1805 | 7A | 42.5 | 6.30E-01 | 2.20E-04 | 1.10E-02 | 1.10E-02 | 2.10E-03 | 5.40E-01 | 6.50E-02 |
| IWA2929 |  | 172.5 | 4.20E-03 | 3.10E-04 | 1.60E-03 | 6.20E-04 | 9.80E-03 | 8.20E-02 | 7.30E-02 |
| IWA7728 |  | 173.2 | 5.10E-01 | 9.80E-04 | 9.60E-02 | 7.10E-02 | 8.80E-02 | 1.30E-01 | 1.90E-01 |
| IWA3371 |  | 175.6 | 4.90E-03 | 1.30E-04 | 4.70E-03 | 3.90E-03 | 2.50E-02 | 1.50E-01 | 2.70E-02 |
| IWA4175 |  | 175.6 | 6.80E-04 | 1.10E-01 | 4.10E-02 | 7.90E-01 | 3.90E-01 | 4.60E-02 | 4.40E-02 |
| IWA2522 | 7D | 94.7 | 8.30E-03 | 8.90E-02 | 7.90E-03 | 6.90E-04 | 1.90E-02 | 1.60E-02 | 2.20E-02 |
| IWA2524 |  | 94.7 | 1.20E-02 | 3.10E-02 | 4.30E-03 | 1.00E-03 | 4.70E-02 | 4.80E-03 | 2.90E-02 |
| IWA266 |  | 98.1 | 3.10E-03 | 2.40E-02 | 4.1E-05^**^ | 2.3E-05^**^ | 3.30E-04 | 1.10E-02 | 1.90E-03 |

^a^SNP, chromosome and position information was derived from Cavanagh et al., 2013

^*,**^*P* value significant at FDR 0.1 or Bonferroni 0.1, respectively

**Reference:** Cavanagh C, Chao S, Wang S, Huang BE, Stephen S. Genome-wide comparative diversity uncovers multiple targets of selection for improvement in hexaploid wheat landraces and cultivars. Proc Natl Acad Sci USA. 2013;110: 8057–8062.
