# Supplementary material for: Genome-wide associations for multiple pest resistances in a Northwestern United States elite spring wheat panel
Source: PLoS One. 2018 Feb 7;13(2):e0191305. doi: 10.1371/journal.pone.0191305 (PMC5802848; doi:10.1371/journal.pone.0191305)
Supplement: S4 Table — (DOCX) [file pone.0191305.s007.docx]

**S4 Table**. **Chromosome location and *R^2^* values of significantly associated SNP markers at unique stage(s) and disease phenotype.**

|  |  |  | **Seedling IT** | | | **Adult IT** | | | | | | | **Adult SEV** | | | | | | |
| --- | --- | --- | --- | --- | --- | --- | --- | --- | --- | --- | --- | --- | --- | --- | --- | --- | --- | --- | --- |
| **Marker** | **Chr^a^.** | **Pos^a^.** | **SP11** | **MV12** | **MV13** | **SP11** | **SP12** | **SP13** | **WL12** | **WL13** | **MV12** | **MV13** | **SP11** | **SP12** | **SP13** | **WL12** | **WL13** | **MV12** | **MV13** |
| **Seedling IT** | |  |  |  |  |  |  |  |  |  |  |  |  |  |  |  |  |  |  |
| IWA8557 | 1B | 18.7 | 0.5 | 1.6 | 3.3 | 0.0 | 1.5 | 1.2 | 1.0 | 0.9 | 0.5 | 0.5 | 0.8 | 0.3 | 0.3 | 0.7 | 1.2 | 1.5 | 0.1 |
| IWA3620 |  | 33.4 | 0.1 | 0.5 | 3.0 | 0.0 | 0.8 | 1.8 | 0.3 | 1.2 | 1.7 | 1.2 | 0.5 | 0.3 | 0.8 | 1.0 | 0.3 | 0.6 | 0.4 |
| IWA4975 |  | 33.4 | 0.2 | 0.5 | 3.0 | 0.1 | 0.4 | 1.2 | 0.1 | 1.3 | 1.1 | 1.1 | 0.4 | 0.2 | 0.5 | 0.8 | 0.5 | 0.6 | 0.3 |
| IWA7737 |  | 33.4 | 0.2 | 0.5 | 3.4 | 0.1 | 0.5 | 1.7 | 0.2 | 1.6 | 1.3 | 1.3 | 0.6 | 0.3 | 0.5 | 0.9 | 0.5 | 0.7 | 0.4 |
| IWA8599 | 2B | 70.2 | 2.7 | 1.8 | 1.2 | 1.2 | 0.7 | 0.0 | 0.4 | 0.9 | 0.1 | 0.6 | 0.6 | 0.9 | 0.0 | 0.0 | 0.8 | 0.2 | 0.2 |
| IWA2873 |  | 202.2 | 4.2 | 0.1 | 0.2 | 0.3 | 1.6 | 0.4 | 0.5 | 0.5 | 0.9 | 0.0 | 0.7 | 0.6 | 0.8 | 0.3 | 0.8 | 0.3 | 0.2 |
| IWA3176 |  | 202.0 | 4.0 | 0.0 | 0.1 | 0.3 | 1.6 | 0.3 | 0.5 | 0.6 | 0.5 | 0.0 | 1.2 | 1.4 | 1.1 | 0.3 | 0.9 | 0.3 | 0.4 |
| IWA7640 |  | 210.2 | 2.6 | 0.1 | 0.9 | 0.1 | 1.2 | 0.6 | 0.3 | 0.8 | 0.5 | 0.6 | 0.6 | 1.2 | 1.6 | 0.3 | 0.5 | 1.1 | 0.5 |
| IWA7009 | 5A | 166.4 | 0.1 | 3.0 | 0.2 | 0.0 | 0.0 | 0.1 | 0.7 | 0.2 | 0.0 | 0.0 | 0.2 | 0.2 | 0.0 | 0.3 | 0.0 | 0.0 | 0.0 |
| IWA6902 | 5B | 205.0 | 3.9 | 0.6 | 1.8 | 0.0 | 0.1 | 0.7 | 0.8 | 0.1 | 0.2 | 0.8 | 0.4 | 0.0 | 0.2 | 0.0 | 0.0 | 0.1 | 0.3 |
|  |  |  |  |  |  |  |  |  |  |  |  |  |  |  |  |  |  |  |  |
| **Adult IT** | |  |  |  |  |  |  |  |  |  |  |  |  |  |  |  |  |  |  |
| IWA7578 | 1B | 26.3 | 0.5 | 1.4 | 0.7 | 0.5 | 1.3 | 1.0 | 0.9 | 1.2 | 2.5 | 1.1 | 0.2 | 0.8 | 1.3 | 1.1 | 0.5 | 0.7 | 1.7 |
| IWA2504 |  | 47.5 | 0.4 | 0.0 | 0.0 | 3.4 | 0.4 | 1.2 | 1.2 | 0.8 | 0.1 | 1.5 | 1.9 | 0.1 | 1.3 | 0.7 | 0.8 | 0.1 | 1.1 |
| IWA3348 |  | 47.5 | 0.4 | 0.1 | 0.0 | 3.1 | 0.6 | 1.2 | 0.9 | 1.4 | 0.1 | 1.5 | 2.0 | 0.5 | 1.1 | 1.1 | 0.8 | 0.2 | 0.9 |
| IWA5418 |  | 47.5 | 0.2 | 0.2 | 0.2 | 2.0 | 0.2 | 0.1 | 0.1 | 0.1 | 0.0 | 0.7 | 0.7 | 0.0 | 0.3 | 0.2 | 0.1 | 0.0 | 0.1 |
| IWA7466 |  | 47.5 | 0.2 | 0.3 | 0.2 | 2.0 | 0.2 | 0.1 | 0.2 | 0.1 | 0.0 | 0.9 | 0.6 | 0.0 | 0.3 | 0.2 | 0.1 | 0.0 | 0.2 |
| IWA7017 |  | 56.7 | 0.4 | 0.8 | 0.3 | 2.0 | 1.1 | 1.9 | 0.5 | 0.9 | 0.1 | 1.1 | 0.9 | 0.5 | 1.8 | 0.5 | 0.6 | 0.2 | 0.6 |
| IWA7876 | 2A | 30.5 | 0.0 | 0.3 | 0.5 | 0.5 | 0.5 | 0.4 | 2.2 | 0.6 | 0.6 | 0.0 | 1.0 | 0.0 | 1.0 | 0.3 | 0.1 | 0.6 | 0.1 |
| IWA4866 | 2B | 194.8 | 0.9 | 0.1 | 0.2 | 2.5 | 0.9 | 0.9 | 0.1 | 0.4 | 0.4 | 0.1 | 1.8 | 2.2 | 0.6 | 0.5 | 0.3 | 1.2 | 1.0 |
| IWA3148 |  | 200.1 | 2.3 | 2.3 | 1.6 | 1.7 | 2.9 | 1.7 | 0.9 | 1.6 | 0.9 | 1.1 | 2.1 | 1.9 | 0.6 | 1.1 | 1.0 | 1.6 | 1.6 |
| IWA3103 | 3B | 4.2 | 0.4 | 0.2 | 0.1 | 2.8 | 1.3 | 0.8 | 0.9 | 0.3 | 0.6 | 0.4 | 1.4 | 1.2 | 0.4 | 0.3 | 0.0 | 1.1 | 0.7 |
| IWA5201 |  | 4.2 | 0.0 | 0.1 | 0.3 | 2.0 | 0.5 | 0.1 | 0.2 | 0.3 | 0.3 | 0.9 | 0.3 | 0.8 | 0.3 | 0.0 | 0.1 | 1.8 | 1.2 |
| IWA5202 |  | 3.9 | 0.3 | 0.4 | 0.0 | 2.7 | 1.0 | 0.6 | 0.8 | 0.3 | 0.5 | 0.3 | 1.3 | 0.7 | 0.3 | 0.1 | 0.0 | 0.6 | 0.5 |
| IWA5203 |  | 3.7 | 0.3 | 0.0 | 0.0 | 2.9 | 1.4 | 0.7 | 0.8 | 0.3 | 0.3 | 0.5 | 1.0 | 1.2 | 0.4 | 0.2 | 0.0 | 1.5 | 0.7 |
| IWA1798 | 4B | 124.3 | 0.4 | 0.0 | 0.0 | 2.2 | 0.8 | 0.4 | 0.9 | 0.6 | 0.4 | 0.2 | 0.7 | 0.4 | 1.7 | 0.6 | 0.1 | 0.0 | 1.0 |
| IWA2087 |  | 124.3 | 0.3 | 0.1 | 0.0 | 2.3 | 0.6 | 0.2 | 0.9 | 0.2 | 0.3 | 0.1 | 0.4 | 0.5 | 1.4 | 0.4 | 0.0 | 0.0 | 0.9 |
| IWA2469 |  | 124.3 | 0.2 | 0.1 | 0.0 | 2.4 | 0.8 | 0.8 | 0.7 | 0.6 | 0.6 | 0.3 | 1.3 | 0.6 | 1.9 | 0.7 | 0.1 | 0.1 | 1.3 |
| IWA2470 |  | 124.3 | 0.2 | 0.0 | 0.1 | 2.5 | 1.4 | 0.7 | 0.8 | 0.5 | 0.4 | 0.4 | 1.5 | 0.5 | 1.8 | 0.8 | 0.1 | 0.0 | 0.7 |
| IWA4615 |  | 124.5 | 0.3 | 0.1 | 0.0 | 2.4 | 1.2 | 0.8 | 0.9 | 0.7 | 0.7 | 0.3 | 0.9 | 0.7 | 2.0 | 0.7 | 0.2 | 0.1 | 1.0 |
| IWA4618 |  | 124.5 | 0.1 | 0.0 | 0.1 | 2.2 | 1.0 | 0.4 | 0.9 | 0.7 | 0.3 | 0.4 | 0.4 | 0.4 | 1.7 | 0.6 | 0.2 | 0.1 | 0.8 |
| IWA7299 |  | 124.9 | 0.3 | 0.0 | 0.0 | 2.5 | 0.8 | 0.4 | 1.0 | 0.4 | 0.4 | 0.3 | 0.5 | 0.5 | 1.7 | 0.5 | 0.1 | 0.0 | 1.0 |
| IWA2145 | 5A | 12.9 | 0.3 | 0.1 | 0.3 | 0.1 | 0.2 | 0.2 | 0.4 | 0.9 | 0.1 | 2.2 | 0.5 | 0.1 | 0.0 | 0.3 | 0.1 | 0.3 | 0.9 |
| IWA2146 |  | 12.9 | 0.6 | 0.2 | 0.4 | 0.2 | 0.1 | 0.1 | 0.6 | 0.9 | 0.0 | 2.3 | 0.3 | 0.1 | 0.0 | 0.3 | 0.1 | 0.6 | 1.4 |
| IWA6405 |  | 36.8 | 0.2 | 0.6 | 1.8 | 0.1 | 1.4 | 0.7 | 2.4 | 1.4 | 0.5 | 0.1 | 1.4 | 0.2 | 0.2 | 0.5 | 0.2 | 0.1 | 0.1 |
| IWA1280 |  | 54.2 | 1.3 | 0.4 | 0.4 | 2.8 | 1.6 | 1.9 | 0.6 | 1.9 | 0.8 | 3.0 | 0.6 | 0.5 | 1.1 | 1.1 | 1.2 | 0.4 | 2.1 |
| IWA2282 |  | 162.9 | 0.3 | 0.9 | 0.7 | 0.0 | 1.4 | 0.3 | 0.6 | 0.5 | 2.3 | 0.3 | 1.1 | 0.2 | 0.6 | 0.4 | 1.3 | 1.4 | 0.2 |
| IWA5950 | 5B | 152.4 | 0.0 | 0.4 | 0.7 | 0.6 | 0.4 | 2.3 | 1.9 | 2.4 | 0.2 | 0.9 | 1.1 | 0.0 | 2.1 | 1.4 | 1.1 | 0.5 | 0.8 |
| IWA2255 |  | 152.8 | 0.4 | 0.2 | 0.2 | 1.0 | 0.7 | 2.3 | 1.6 | 1.1 | 0.9 | 1.4 | 1.1 | 0.2 | 0.3 | 0.9 | 0.4 | 0.2 | 1.0 |
| IWA1427 | 5D | 0.0 | 0.8 | 0.0 | 1.6 | 1.5 | 2.8 | 1.4 | 1.0 | 1.6 | 0.9 | 0.6 | 1.3 | 0.9 | 1.1 | 1.6 | 1.2 | 1.1 | 1.6 |
| IWA1428 |  | 0.0 | 0.7 | 0.0 | 1.5 | 1.5 | 2.7 | 1.4 | 0.9 | 1.4 | 1.0 | 0.5 | 1.5 | 1.0 | 1.1 | 1.5 | 1.2 | 1.2 | 1.4 |
| IWA1429 |  | 0.0 | 0.7 | 0.0 | 1.2 | 1.4 | 2.4 | 1.4 | 0.9 | 1.6 | 0.9 | 0.7 | 1.5 | 1.0 | 1.2 | 1.6 | 1.2 | 0.9 | 1.5 |
| IWA1493 | 6B | 0.6 | 0.1 | 0.1 | 0.1 | 2.4 | 0.2 | 0.4 | 0.0 | 0.3 | 0.0 | 0.0 | 1.2 | 0.2 | 0.4 | 0.2 | 0.0 | 0.0 | 0.0 |
| IWA1531 |  | 96.3 | 0.3 | 0.3 | 0.1 | 1.9 | 1.2 | 0.8 | 0.6 | 0.1 | 0.1 | 0.4 | 1.5 | 1.0 | 0.5 | 1.0 | 0.7 | 0.0 | 0.1 |
| IWA6507 | 7A | 40.6 | 1.1 | 0.1 | 0.3 | 0.8 | 0.6 | 0.5 | 0.0 | 1.0 | 2.9 | 1.0 | 1.1 | 0.0 | 0.0 | 0.3 | 1.6 | 0.5 | 1.0 |
| IWA5390 | 7B | 19.8 | 0.2 | 0.6 | 0.6 | 0.4 | 0.4 | 0.5 | 0.8 | 1.1 | 0.2 | 2.7 | 0.1 | 0.0 | 1.2 | 0.1 | 0.3 | 0.6 | 0.6 |
| IWA4857 |  | 100.4 | 1.1 | 1.1 | 1.8 | 0.4 | 0.7 | 1.8 | 0.6 | 2.2 | 1.1 | 1.6 | 1.0 | 1.8 | 0.5 | 1.4 | 2.1 | 0.4 | 0.6 |
|  |  |  |  |  |  |  |  |  |  |  |  |  |  |  |  |  |  |  |  |
| **Adult SEV** | |  |  |  |  |  |  |  |  |  |  |  |  |  |  |  |  |  |  |
| IWA6889 | 1B | 21.7 | 0.3 | 0.0 | 0.4 | 0.1 | 0.4 | 0.8 | 0.9 | 1.3 | 1.2 | 0.9 | 0.8 | 1.3 | 1.2 | 1.1 | 1.1 | 2.8 | 1.0 |
| IWA4678 |  | 22.5 | 0.7 | 0.3 | 0.2 | 0.0 | 0.2 | 0.8 | 0.6 | 1.7 | 1.6 | 0.4 | 0.6 | 0.4 | 0.9 | 1.2 | 1.6 | 2.6 | 0.8 |
| IWA3143 | 2A | 5.9 | 0.2 | 0.3 | 0.7 | 0.3 | 0.7 | 1.0 | 1.2 | 1.7 | 0.8 | 1.9 | 1.0 | 1.1 | 1.7 | 0.5 | 1.7 | 2.6 | 1.8 |
| IWA4098 | 2B | 197.7 | 1.2 | 0.1 | 0.0 | 1.2 | 0.7 | 0.3 | 0.3 | 0.3 | 0.8 | 0.5 | 3.1 | 1.8 | 0.6 | 2.4 | 0.5 | 1.2 | 1.1 |
| IWA2676 |  | 200.1 | 2.3 | 1.1 | 0.9 | 1.8 | 1.8 | 1.4 | 0.7 | 1.3 | 0.9 | 0.4 | 2.5 | 2.0 | 0.9 | 1.8 | 1.1 | 1.9 | 1.1 |
| IWA4097 |  | 200.5 | 0.8 | 0.1 | 0.0 | 0.9 | 0.6 | 0.2 | 0.2 | 0.2 | 0.3 | 0.5 | 2.8 | 2.1 | 0.5 | 1.5 | 0.4 | 0.9 | 1.2 |
| IWA7113 |  | 219.9 | 0.0 | 0.4 | 0.4 | 0.2 | 1.3 | 0.3 | 0.5 | 0.3 | 0.6 | 0.2 | 0.1 | 0.3 | 2.2 | 0.4 | 0.3 | 0.9 | 0.5 |
| IWA3594 |  | 249.5 | 1.3 | 0.3 | 0.3 | 1.4 | 2.0 | 1.3 | 0.6 | 0.4 | 0.8 | 0.2 | 2.3 | 0.3 | 0.3 | 0.3 | 0.3 | 0.3 | 0.4 |
| IWA1617 | 3B | 75.7 | 0.0 | 0.5 | 0.0 | 0.0 | 0.1 | 0.1 | 0.3 | 0.4 | 0.4 | 0.1 | 0.1 | 0.5 | 0.2 | 1.1 | 0.8 | 2.7 | 0.3 |
| IWA8058 |  | 165.8 | 0.0 | 0.7 | 0.0 | 0.6 | 0.2 | 0.2 | 0.4 | 0.9 | 0.0 | 1.7 | 0.1 | 0.2 | 1.4 | 0.7 | 1.1 | 1.2 | 2.2 |
| IWA1992 | 4A | 44.0 | 0.0 | 0.0 | 0.0 | 0.4 | 0.6 | 1.2 | 0.3 | 0.1 | 0.9 | 0.0 | 0.5 | 2.5 | 0.1 | 0.5 | 0.0 | 1.4 | 0.4 |
| IWA2170 |  | 167.3 | 0.5 | 0.1 | 0.2 | 0.4 | 1.3 | 1.3 | 0.7 | 1.1 | 0.1 | 0.5 | 0.2 | 2.4 | 0.7 | 1.3 | 1.3 | 0.5 | 0.5 |
| IWA1062 | 5A | 31.4 | 0.3 | 0.1 | 0.5 | 0.3 | 0.9 | 0.6 | 0.3 | 1.3 | 0.6 | 0.7 | 0.6 | 0.3 | 0.4 | 0.3 | 2.2 | 1.0 | 0.8 |
| IWA3989 |  | 112.0 | 0.1 | 0.6 | 0.9 | 0.0 | 0.4 | 0.8 | 0.6 | 1.6 | 0.5 | 1.1 | 0.9 | 0.1 | 0.5 | 0.7 | 2.5 | 1.1 | 0.2 |
| IWA3990 |  | 112.0 | 0.0 | 0.5 | 0.9 | 0.1 | 0.5 | 0.9 | 0.8 | 1.8 | 0.5 | 1.2 | 1.0 | 0.1 | 0.5 | 0.7 | 2.3 | 1.1 | 0.2 |
| IWA6988 |  | 190.4 | 0.1 | 0.7 | 0.8 | 0.4 | 0.3 | 1.1 | 1.0 | 2.0 | 0.2 | 1.2 | 0.7 | 0.0 | 0.6 | 1.2 | 2.7 | 1.1 | 1.2 |
| IWA7815 | 5B | 121.8 | 0.5 | 1.2 | 0.9 | 0.1 | 0.8 | 1.3 | 1.2 | 1.7 | 0.5 | 0.9 | 0.9 | 0.9 | 3.2 | 0.8 | 1.5 | 2.5 | 1.2 |
| IWA584 |  | 172.5 | 0.1 | 0.0 | 0.5 | 0.7 | 0.7 | 0.8 | 1.3 | 0.9 | 0.1 | 1.5 | 2.3 | 0.0 | 1.1 | 0.6 | 0.9 | 0.0 | 1.2 |
| IWA1805 | 7A | 42.5 | 0.5 | 0.0 | 0.1 | 0.7 | 0.5 | 0.5 | 0.3 | 0.1 | 0.9 | 1.2 | 0.0 | 3.0 | 1.3 | 0.1 | 0.7 | 1.5 | 1.8 |
| IWA2929 |  | 172.5 | 0.0 | 0.0 | 0.1 | 0.5 | 0.4 | 1.0 | 0.4 | 0.6 | 1.0 | 0.7 | 1.7 | 2.8 | 2.0 | 0.6 | 0.6 | 2.8 | 1.3 |
| IWA7728 |  | 173.2 | 0.0 | 0.2 | 0.7 | 0.9 | 0.8 | 1.0 | 0.5 | 0.6 | 1.1 | 0.2 | 0.1 | 2.4 | 0.6 | 0.4 | 0.3 | 0.8 | 0.6 |
| IWA3371 |  | 175.6 | 0.0 | 0.1 | 0.4 | 1.4 | 1.7 | 1.6 | 1.0 | 0.9 | 0.4 | 1.3 | 1.6 | 3.2 | 1.6 | 0.4 | 1.0 | 2.0 | 1.0 |
| IWA4175 |  | 175.6 | 0.2 | 0.0 | 0.1 | 0.4 | 0.3 | 0.3 | 0.4 | 0.6 | 0.2 | 1.3 | 2.4 | 0.5 | 0.8 | 0.8 | 0.8 | 0.0 | 0.1 |
| IWA2522 | 7D | 94.7 | 0.4 | 0.4 | 0.1 | 0.8 | 0.7 | 0.2 | 0.8 | 1.3 | 0.5 | 1.2 | 1.4 | 0.6 | 1.4 | 1.1 | 1.0 | 2.7 | 1.1 |
| IWA2524 |  | 94.7 | 0.2 | 0.6 | 0.0 | 0.6 | 0.6 | 0.4 | 0.5 | 1.3 | 0.2 | 1.2 | 1.3 | 1.0 | 1.6 | 1.5 | 0.9 | 2.5 | 0.8 |
| IWA266 |  | 98.1 | 0.2 | 0.3 | 0.6 | 0.8 | 1.1 | 0.9 | 0.6 | 1.9 | 0.7 | 1.4 | 1.8 | 1.1 | 3.4 | 1.2 | 1.9 | 4.3 | 2.5 |
|  |  |  |  |  |  |  |  |  |  |  |  |  |  |  |  |  |  |  |  |
| **Adult IT and SEV** | | |  |  |  |  |  |  |  |  |  |  |  |  |  |  |  |  |  |
| IWA2179 | 2B | 194.8 | 1.3 | 0.0 | 0.1 | 2.3 | 1.4 | 1.1 | 0.3 | 0.7 | 0.5 | 0.1 | 2.7 | 2.3 | 0.8 | 0.7 | 0.4 | 1.4 | 1.1 |
| IWA3938 |  | 211.1 | 0.8 | 1.0 | 1.4 | 1.3 | 1.0 | 0.9 | 2.7 | 0.9 | 1.9 | 2.3 | 1.7 | 2.5 | 1.6 | 1.3 | 1.3 | 2.1 | 1.3 |
| IWA8266 |  | 211.8 | 2.0 | 0.0 | 0.1 | 0.7 | 2.6 | 0.4 | 1.4 | 1.5 | 0.9 | 0.7 | 2.4 | 2.9 | 1.1 | 2.1 | 1.6 | 0.6 | 1.6 |
| IWA4796 | 3B | 1.9 | 0.5 | 0.5 | 0.8 | 2.5 | 1.4 | 0.8 | 0.6 | 0.5 | 1.8 | 0.6 | 1.0 | 1.6 | 0.3 | 0.4 | 0.3 | 3.5 | 1.0 |
| IWA7989 | 5B | 156.7 | 0.2 | 0.0 | 0.3 | 1.0 | 0.5 | 1.2 | 3.1 | 0.8 | 0.7 | 1.7 | 0.8 | 0.4 | 1.3 | 1.0 | 0.0 | 0.2 | 2.3 |
|  |  |  |  |  |  |  |  |  |  |  |  |  |  |  |  |  |  |  |  |
| **Seedling IT and adult SEV** | | |  |  |  |  |  |  |  |  |  |  |  |  |  |  |  |  |  |
| IWA2379 | 2B | 195.8 | 3.3 | 0.3 | 0.1 | 1.1 | 1.2 | 0.9 | 0.5 | 0.9 | 0.8 | 0.2 | 3.7 | 2.0 | 1.0 | 1.9 | 1.2 | 0.6 | 0.3 |
| IWA2678 |  | 196.8 | 3.0 | 0.5 | 0.5 | 1.6 | 1.7 | 1.3 | 0.5 | 1.1 | 1.0 | 0.1 | 2.3 | 1.8 | 0.9 | 2.0 | 0.9 | 1.4 | 0.7 |
| IWA2701 |  | 195.8 | 4.2 | 0.5 | 0.3 | 1.7 | 1.3 | 1.1 | 0.7 | 0.8 | 1.1 | 0.2 | 4.3 | 2.2 | 1.1 | 1.9 | 1.2 | 1.2 | 0.8 |
|  |  |  |  |  |  |  |  |  |  |  |  |  |  |  |  |  |  |  |  |
| **Seedling IT and adult IT** | | |  |  |  |  |  |  |  |  |  |  |  |  |  |  |  |  |  |
| IWA2577 | 1B | 23.7 | 0.5 | 1.2 | 3.7 | 0.6 | 2.1 | 3.1 | 2.9 | 1.9 | 1.4 | 1.6 | 1.0 | 2.1 | 1.1 | 1.7 | 1.5 | 0.3 | 0.3 |
| IWA4504 |  | 40.4 | 0.9 | 1.2 | 2.5 | 0.4 | 1.2 | 1.4 | 1.9 | 2.0 | 2.6 | 0.9 | 1.0 | 1.1 | 0.4 | 1.6 | 1.9 | 1.2 | 1.2 |
| IWA6449 |  | 40.4 | 0.7 | 1.0 | 2.5 | 0.3 | 1.4 | 1.4 | 2.2 | 2.2 | 2.4 | 1.2 | 1.0 | 1.1 | 0.4 | 2.0 | 2.0 | 1.3 | 1.4 |
| IWA7638 | 2A | 243.8 | 0.4 | 1.2 | 2.7 | 1.1 | 1.9 | 1.8 | 1.2 | 1.6 | 2.2 | 1.5 | 0.7 | 1.1 | 0.7 | 1.0 | 0.6 | 1.1 | 2.1 |

^a^SNP chromosome and position information was derived from Cavanagh et al., 2013

**Reference:** Cavanagh C, Chao S, Wang S, Huang BE, Stephen S. Genome-wide comparative diversity uncovers multiple targets of selection for improvement in hexaploid wheat landraces and cultivars. Proc Natl Acad Sci USA. 2013;110: 8057–8062
