# Supplementary material for: Genome-wide associations for multiple pest resistances in a Northwestern United States elite spring wheat panel
Source: PLoS One. 2018 Feb 7;13(2):e0191305. doi: 10.1371/journal.pone.0191305 (PMC5802848; doi:10.1371/journal.pone.0191305)
Supplement: S5 Table — (DOCX) [file pone.0191305.s008.docx]

**S5 Table.** **Analysis of variance (ANOVA) for disease severity (%) response to *Septoria* *tritici* blotch in Holeta and Bekoji, Ethiopia.**

|  | **% Severity** |
| --- | --- |
| ***σ*^2^*_G_*** | 144.7^***^ |
| ***σ*^2^*_E_*** | 1499.4^ns^ |
| ***σ*^2^*_GE_*** | 193.9^***^ |
| ***σ*^2^*_e_*** | 1.1^ns^ |
| ***H^2^*** | 0.2 |

^ns,*,**,***^ Pearson’s correlation nonsignificant, significant at *P ≤* 0.05, 0.01, or 0.001*,* respectively
